# Patatin-like phospholipase CapV in *Escherichia coli* - morphological and physiological effects of one amino acid substitution

**DOI:** 10.1038/s41522-022-00294-z

**Published:** 2022-05-11

**Authors:** Fengyang Li, Lianying Cao, Heike Bähre, Soo-Kyoung Kim, Kristen Schroeder, Kristina Jonas, Kira Koonce, Solomon A. Mekonnen, Soumitra Mohanty, Fengwu Bai, Annelie Brauner, Vincent T. Lee, Manfred Rohde, Ute Römling

**Affiliations:** 1 Department of Microbiology, Tumor and Cell Biology, Karolinska Institutet, 17177 Stockholm, Sweden; 2 Research Core Unit Metabolomics, Hannover Medical School, Hannover, Germany; 3 Department of Cell Biology and Molecular Genetics, University of Maryland, College Park, MD 20742 USA; 4 Science for Life Laboratory, Department of Molecular Biosciences, The Wenner-Gren Institute, Stockholm University, Stockholm, Sweden; 5 Department of Microbiology, Tumor and Cell Biology, Division of Clinical Microbiology, Karolinska Institutet and Karolinska University Hospital, 17176 Stockholm, Sweden; 6 School of Life Sciences and Biotechnology, Shanghai Jiao Tong University, 200240 Shanghai, China; 7 Central Facility for Microscopy, Helmholtz Center for Infection Research, Braunschweig, Germany; 8 Present Address: College of Veterinary Medicine, Jilin University, Changchun, China

**Keywords:** Microbial ecology, Molecular evolution

## Abstract

In rod-shaped bacteria, morphological plasticity occurs in response to stress, which blocks cell division to promote filamentation. We demonstrate here that overexpression of the patatin-like phospholipase variant CapV_Q329R_, but not CapV, causes pronounced *sulA*-independent pyridoxine-inhibited cell filamentation in the *Escherichia coli* K-12-derivative MG1655 associated with restriction of flagella production and swimming motility. Conserved amino acids in canonical patatin-like phospholipase A motifs, but not the nucleophilic serine, are required to mediate CapV_Q329R_ phenotypes. Furthermore, CapV_Q329R_ production substantially alters the lipidome and colony morphotype including rdar biofilm formation with modulation of the production of the biofilm activator CsgD, and affects additional bacterial traits such as the efficiency of phage infection and antimicrobial susceptibility. Moreover, genetically diverse commensal and pathogenic *E. coli* strains and *Salmonella typhimurium* responded with cell filamentation and modulation in colony morphotype formation to CapV_Q329R_ expression. In conclusion, this work identifies the CapV variant CapV_Q329R_ as a pleiotropic regulator, emphasizes a scaffold function for patatin-like phospholipases, and highlights the impact of the substitution of a single conserved amino acid for protein functionality and alteration of host physiology.

## Introduction

Bacteria are defined by their cell shape such as rod, coccus, spiral or filamentous, which exist as unicellular or multinucleated cells^1^. Shaped differently, many bacterial species display extensive morphological plasticity in response to environmental cues including severe stress^2,3^. Such morphological variation is often reversible, suggesting an altered physiological cell status or epigenetic modulation of the genetic information upon signal perception or manifested threat. For example, to escape from intracellular biofilms during late infection of bladder cells, rod-shaped uropathogenic *Escherichia coli* (UPEC) can reversibly transform from a rod with a length of 2-4 μm into a filament of up to 70 μm in length^4^. In nature, *Caulobacter crescentus*, a freshwater curved rod-shaped bacterium, can develop 20 μm long helical filaments which escape from biofilms for nutrient acquisition^5,6^. Other types of filamentous bacteria of diverse phylogenetic origin are commonly found in activated sludge and as host-dependent immunomodulatory ‘segmented filamentous bacteria’ in the gut^7,8^. The giant *Epulopiscium fishelsoni* can become up to 750 μm long^9^.

Upon exposure to various environmental stresses including nutrient deficiency^6,10^, antibiotic treatment^11–13^, and DNA damage^14–16^, bacterial cell filamentation is a consequence of activation of the SOS response system. This is accomplished mainly by impairment of the functionality of the essential cell division protein, the tubulin homolog FtsZ, which initiates cell division by forming a septal ring at the prospective invagination site upon polymerization^17–19^. Negative regulation of septum forming FtsZ can be exerted by expression of the SOS factor SulA which interacts with FtsZ to inhibit polymerization eventually leading to cell filamentation^18,20–22^. The subsequent arrest in growth enables the cells to recover from otherwise deleterious damages before resuming growth. However, there exist alternative SOS responses and *sulA*-independent stress-associated pathways that promote filamentation in *E. coli*. For example, upon treatment with cationic antimicrobial peptides, QueE, an enzyme involved in the queuosine tRNA modification pathway, blocks cell division and induces filamentation^23^.

Temporal emergence of filamentation can serve alternative purposes such as being a survival strategy contributing to pathogenesis. *E. coli* filamentation triggered by treatment with cell wall inhibiting β-lactam antibiotics in patients can promote bacterial surface colonization^24,25^. Filamentation also slows down phagocytosis of UPEC *E. coli* by macrophages during infection to enhance bacterial survival upon challenge by the host immune responses^4,20,26^.

During cell division, cell elongation, chromosome replication and segregation, and cytokinesis (cell separation) are coordinated^27–29^ though dysregulation of critical components and regulators promotes cell filamentation and filament formation^28,30–33^. For example, overexpression of the cell division inhibitor MinC, which prevents FtsZ septum formation at the cell poles by oscillation from pole to pole assisted by MinDE leads to filamentation^34^. DamX, a membrane-spanning protein with a peptidoglycan binding SPOR domain, is required for reversible filamentation, colonization and pathogenesis of UPEC morphotype switching^35^.

Motility is defined as the ability to actively move in liquid or on surfaces^36^. Various bacterial modes of movement are dependent on energetic requirements with diverse structural elements involved^37^. As a common mode of motility, propelling of flagellar filaments moves bacterial cells in liquid medium by swimming motility and on a surface of the semi-solid medium by swarming motility^36,38^. In *E. coli* and the gastrointestinal pathogen *Salmonella enterica* serovar Typhimurium, bacterial motility is tightly regulated by global regulatory signals including the second messenger cyclic di-GMP (c-di-GMP) and protein-protein interactions via enzymatically incompetent EAL domain proteins with catalytically active homologs to be involved in c-di-GMP hydrolysis^38,39^. Cyclic di-GMP is a bacterial intracellular messenger that ubiquitously modulates the single-cell lifestyle transition between motility and sessility for biofilm formation^40,41^. While flagella-based swimming and swarming motility are post-translationally inhibited by c-di-GMP targeting flagella motor functionality^38^, c-di-GMP promotes expression of c*sgD* encoding a major transcriptional biofilm regulator, and subsequently the rdar (red, dry, and rough) colony biofilm morphotype is developed.

Besides the ubiquitous c-di-GMP signaling system, other recently identified cyclic dinucleotide second messengers can modulate sessility/motility lifestyle transition in *E. coli*. The cyclic dinucleotide cyclase DncV synthesizes 3′3′-cyclic AMP-GMP (cAMP-GMP)^42,43^ to subsequently repress motility and rdar biofilm formation in the animal commensal strain *E. coli* ECOR31^43^. *DncV* is part of the putative *capV-dncV-vc0180*-*vc0181* operon located adjacently to the *Yersinia* high-pathogenicity island (HPI)^44,45^. Thereby, *capV* codes for a cAMP-GMP activated patatin-like phospholipase to induce growth retardation and cytolysis in *V. cholerae*^46,47^. Patatin-like phospholipases, which can be found in organisms throughout the phylogenetic tree, hydrolyze the *sn*-2 ester bond of phospholipids and neutral lipids for lipid turnover, remodeling of membrane composition, and signaling to induce programmed cell death^48^. These processes are highly regulated and often involve a co-factor to promote enzymatic activity.

In this study, we show that overexpression of the patatin-like phospholipase variant CapV_Q329R_ causes substantial alteration in cell morphology leading to *sulA*-independent cell filamentation and restricted swimming motility with premature flagella loss. Besides these single-cell morphological changes, which can occur in commensal and pathogenic strains of *E. coli* of diverse genetic background and *S. typhimurium*, CapV_Q329R_ expression altered (rdar) colony biofilm formation and susceptibility to antimicrobials and phage infection. These findings demonstrate that a single amino acid change in a patatin-like phospholipase promotes rapid evolution of protein functionality which leads to the enhanced manipulation of various aspects of bacterial physiology.

## Results

### CapV_Q329R_ inhibits swimming motility of *E. coli* MG1655

**Fig. 1:**
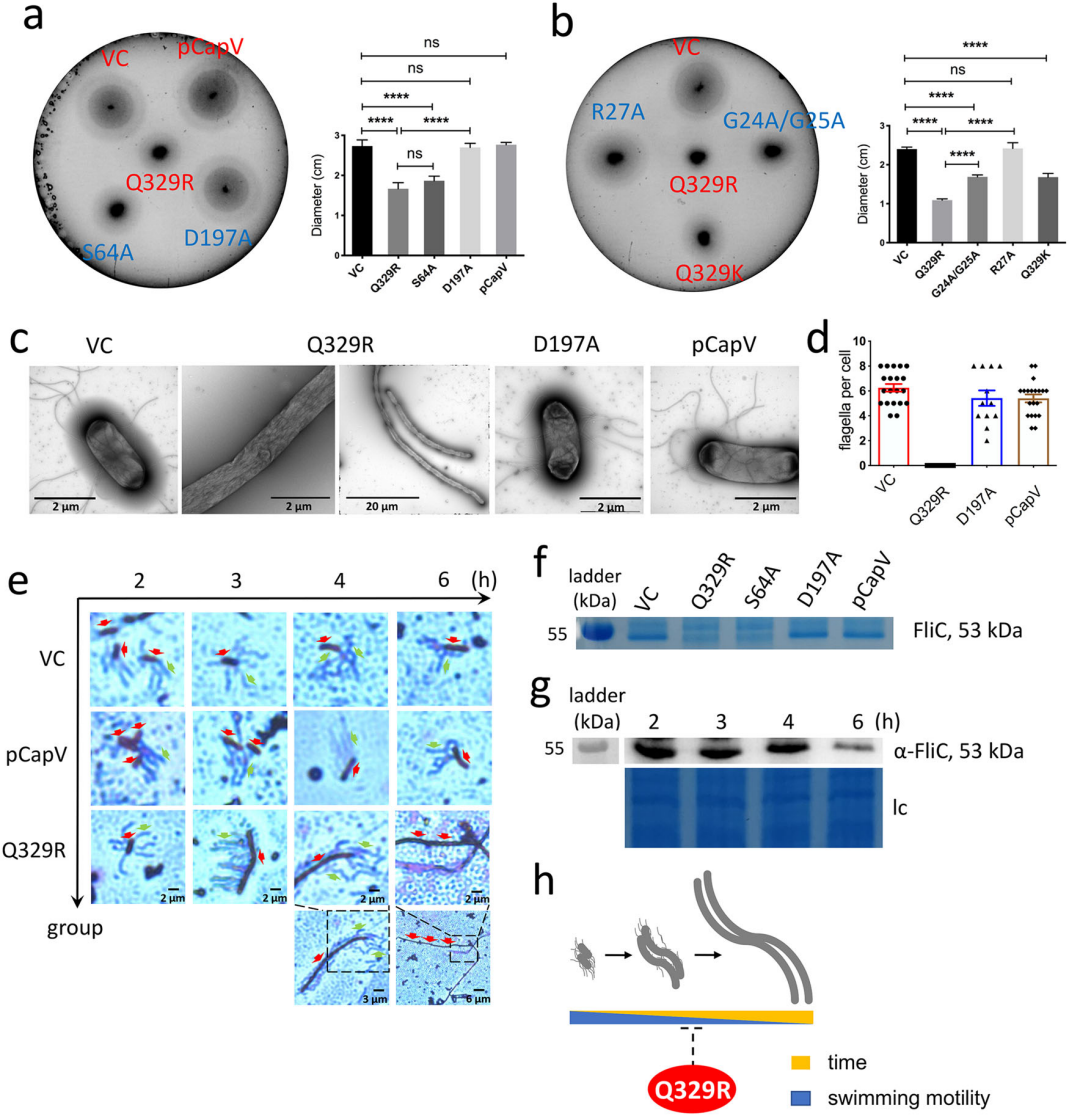
CapV_Q329R_ inhibits apparent swimming motility and production of the flagellin subunit FliC of *E. coli* MG1655. **a**, **b** Flagella-dependent swimming motility of wild-type *E. coli* MG1655 vector control (VC) and upon overexpression of wild-type CapV, its mutants (red) and mutants of CapV_Q329R_ (blue). In total, 3 µl of a OD_600_ = 5 cell suspension were inoculated into soft agar plates containing 1% tryptone, 0.5% NaCl, and 0.25% agar, and the swimming diameter was measured after 6 h at 37 °C. **c** Flagella production of a representative *E. coli* MG1655 VC cell and upon overexpression of CapV, CapV_Q329R_ (Q329R) and CapV_Q329R/D197A_ (D197A) as observed by TEM. **d** Quantification of the number of flagella per cell upon overexpression of CapV and CapV_Q329R_ after visualization by TEM (**c**). The number of evaluated cells *n* = 20. Cells were grown in TB medium for 6 h at 37 °C. **e** Production of surface-associated flagellin of *E. coli* MG1655 VC, upon overexpression of CapV and CapV_Q329R_. **f** Assessment of flagellin subunit FliC expression by colloidal Coomassie staining from *E. coli* MG1655 culture supernatants after shearing of flagella upon expression of CapV, CapV_Q329R_, CapV_Q329R/D197A_ and CapV_Q329R/S64A_. Cells were grown in TB medium at 37 °C for 6 h. **g** Assessment of flagellin subunit FliC expression over time in *E. coli* MG1655 overexpressing CapV_Q329R_. Samples were harvested at different time points in the growth phase for Western blot analysis of FliC. lc, loading control. **h** Proposed development of filamentation and flagella inhibition of *E. coli* MG1655 upon CapV_Q329R_ expression over time. Bars represent mean values from three biologically independent replicates with error bars to represent SD. Differences between mean values were assessed by two-tailed Student’s *t* test: ns, not significant; *P < 0.05, **P < 0.01, and ***P < 0.001 compared to *E. coli* MG1655 VC. Vector control VC = pBAD28. pCapV = CapV cloned in pBAD28; Q329R = CapV_Q329R_ cloned in pBAD28. S64A = CapV _Q329R/S64A_ cloned in pBAD28. D197A = CapV _Q329R/D197A_ cloned in pBAD28. R27A = CapV_Q329R/R27A_ cloned in pBAD28. G24A/G25A = CapV_Q329R/G24A/G25A_ cloned in pBAD28.

The dinucleotide cyclase DncV synthesizes cAMP-GMP to inhibit rdar biofilm formation and motility in the animal commensal strain *E. coli* ECOR31^49^. To assess whether the downstream cAMP-GMP receptor, the patatin-like phospholipase CapV, has a function on its own, we expressed CapV in the heterologous host MG1655, an *E. coli* K-12 derivative which is not known to synthesize cAMP-GMP nor to harbor DncV. Besides wild-type CapV, we overexpressed its variant CapV_Q329R_, which had been derived by cloning a respective mutated DNA fragment as described in Supplementary Results. To this end, we observed that expression of the variant CapV_Q329R_ caused suppression of swimming motility, whereas overexpression of the wild-type protein CapV did not alter the apparent motility in semi-solid tryptone broth (TB) agar (as described in Supplementary Results; Fig. 1a and Supplementary Figs. 1–3). TB medium promotes motility of *E. coli* MG1655 compared to LB medium in the semi-solid agar plate assay^50^. To further characterize CapV_Q329R_-induced *E. coli* MG1655 swimming inhibition, we assessed the production of cell-associated flagella and flagellin upon overexpression of CapV_Q329R_ compared to CapV. Visualization of bacterial cells by transmission electron microscopy (TEM) showed that overexpression of CapV_Q329R_ compared to wild-type CapV dramatically reduced the total number of flagella-producing cells as well as the number of flagella per cell after 6 h incubation at 37 °C (Fig. 1c, d). In agreement, visualization of flagella by Leifson staining upon CapV_Q329R_ overexpression showed cells with intact flagella up to 4 h incubation at 37 °C, but almost no cell with flagella after 6 h (Fig. 1c, e). In congruence with the analysis by TEM and light microscopy examination of Leifson staining, we observed inhibition of production of cell-associated extracellular flagellin in a protein gel upon overexpression of CapV_Q329R_ after 6 h (Fig. 1f, g). Initial analysis of differential gene expression by qRT-PCR indicated 8% downregulation of expression of the class 1 flagella regulon gene *flhD* encoding a subunit of the major regulator FlhD_4_C_2_, and less than 50% downregulation for the representative genes of class 2 *fliA* encoding the flagella specific sigma factor and class 3 *fliC* encoding the subunit of flagella, upon CapV_Q329R_ compared to CapV overexpression. Compared to the vector control, *flhD*, *fliA* and *fliC* were downregulated 28, 83, and 73%, respectively, upon overexpression of CapV. Thus, the expression of CapV_Q329R_ interferes with flagella expression beyond the flagella regulon cascade and can affect, for example, depolymerization or degradation of flagella. Cumulatively, these results indicate that CapV_Q329R_ suppressed swimming motility of MG1655 by post-translationally inhibiting the production of flagellar filaments gradually during the growth phase, while initially production of functional flagellar filaments had been observed.

### CapV_Q329R_ expression promotes cell filamentation of *E. coli* MG1655

Significantly, upon overexpression of CapV_Q329R_ in *E. coli* MG1655 TEM and light microscopy demonstrated not only the loss of flagella, but the concomitant development of long thin filamentous cells (Fig. 1c, e). In contrast, *E. coli* MG1655 cells overexpressing wild-type CapV were only slightly elongated compared to the control (Fig. 1c, e).

**Fig. 2:**
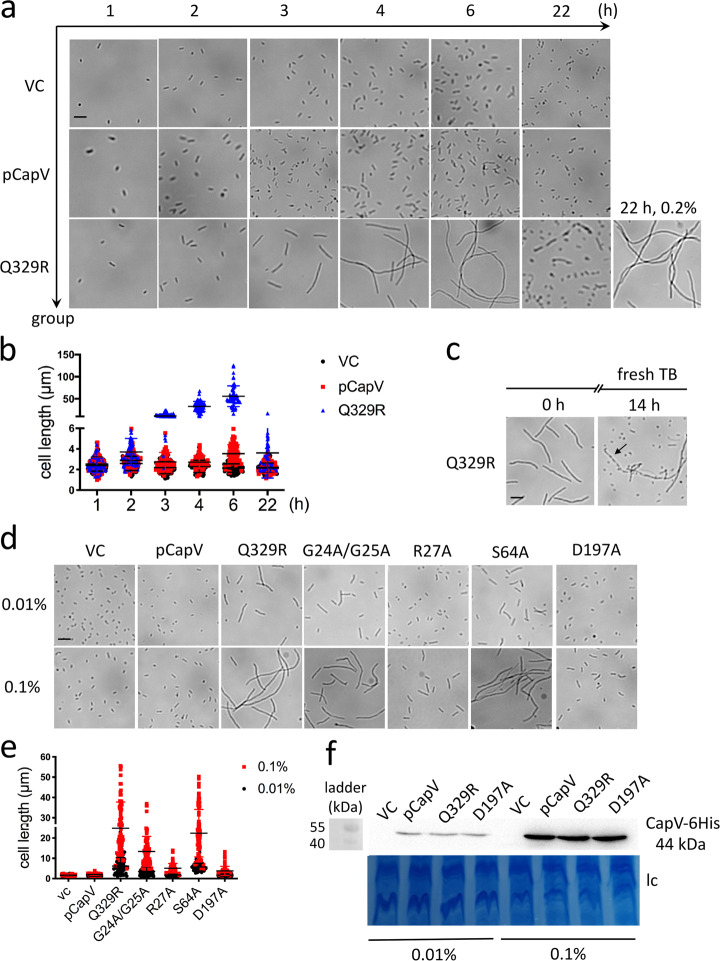
CapV_Q329R_ promotes cell filamentation upon overexpression in *E. coli* MG1655. **a** Light microscopy pictures of cell morphology of *E. coli* MG1655 VC and upon overexpression of CapV and CapV_Q329R_ (Q329R) in TB medium at different time points at 37 °C. **b** Quantification of cell length of *E. coli* MG1655 VC and upon overexpression of CapV and CapV_Q329R_ in TB medium at different time points. The quantification is based on results from at least three independent experiments with the assessment of 70 cells from each group. **c** Cell morphology 3 h after addition of fresh TB medium to filamentous *E. coli* MG1655 cells overexpressing CapV_Q329R_. Arrowheads indicate invaginations at proposed future division sites. **d**, **e**, **f** Assessment of cell length upon overexpression of CapV, CapV_Q329R_ and CapV_Q329R_ derivatives in *E. coli* MG1655 upon induction with different l-arabinose concentrations. Light microscopy pictures (**d**), quantification of cell length (**e**) and protein expression level (lc = loading control) (**f**) upon induction with 0.01% and 0.1% l-arabinose in TB at 37 °C for 4 h. The quantification is based on results from at least three independent experiments with the assessment of 70 cells from each group. Bar, 5 µm. Vector control VC = pBAD28. pCapV = CapV cloned in pBAD28; Q329R = CapV_Q329R_ cloned in pBAD28. G24A/G25A = CapV_Q329R/G24A/G25A_ cloned in pBAD28. R27A = CapV_Q329R/R27A_ cloned in pBAD28. S64A = CapV_Q329R/S64A_ cloned in pBAD28. D197A = CapV_Q329R/D197A_ cloned in pBAD28.

Assessment of the temporal development of cell filamentation throughout the growth phase upon induced expression of CapV_Q329R_ by light microscopy after Leifson staining indicated that filamentation did not initiate before 2 h after commencement of CapV_Q329R_ expression (Figs. 1e and 2a, b). Subsequently, though the cell length and the frequency of filamentation dramatically increased, whereby after 3 h almost all cells displayed as short filaments around 6 times the length of standard rod cells (Fig. 2a, b). After 4 h of induction, CapV_Q329R_ expressing *E. coli* MG1655 cells were on average 25 times longer than control cells. After 6 h of induction, CapV_Q329R_ expressing *E. coli* MG1655 cells were on average more than 50 times longer than the standard rod-shaped cell (Fig. 2, b). Those long cells did not show any movement, while shorter filaments up to approximately 20 times the length of standard *E. coli* cells, although rare, still showed active swimming motility (Supplementary Movie S1). At the opposite, overexpression of wild-type CapV only slightly increased the cell length compared to *E. coli* MG1655 control. On note, after 22 h induction of CapV_Q329R_ expression by 0.1% L-arabinose, short rod-shaped motile cells dominated again, which suggested filaments to restart cell division after CapV_Q329R_ expression had diminished due to L-arabinose depletion. In line with this hypothesis, induction of CapV_Q329R_ production by 0.2% l-arabinose did not cause reversion to rod-shaped cells nor showed the cells any movement after 22 h (Fig. 2a, c and Supplementary Movie S2). Upon transfer of those filamentous cells to fresh TB medium without L-arabinose, however, the emergence of rod-shaped cells was again observed (Fig. 2c). A scheme of this developmental process leading to filamentation with consecutive loss of flagella upon expression of CapV_Q329R_ is displayed in Fig. 1h.

A positive correlation of the filamentation phenotype of the *E. coli* MG1655 cells with CapV_Q329R_ production level was demonstrated using increasing L-arabinose concentrations. When incubated for 4 h with 0.01% l-arabinose, the low-level CapV_Q329R_ expression created a heterogenous cell population displaying no or restricted cell filamentation (Fig. 2d, e). In contrast upon incubation with 0.1% l-arabinose all cells became filamentous as observed previously. Concomitantly, the CapV_Q329R_ expression level was comparable to the CapV expression level excluding that the observed morphological changes were due to significantly different protein expression levels. As expected, CapV expression was higher upon induction with 0.1% l-arabinose than with 0.01% l-arabinose (Fig. 2f). The 6xHis-tag added to the C-terminus of the protein to detect protein production level did not alter the proficiency of CapV_Q329R_ to induce filamentation (Supplementary Fig. 2g). Cumulatively, filamentation and motility repression are caused by the Q320R substitution in CapV.

### CapV is a patatin-like phospholipase, which alters the steady-state lipid profile

**Fig. 3:**
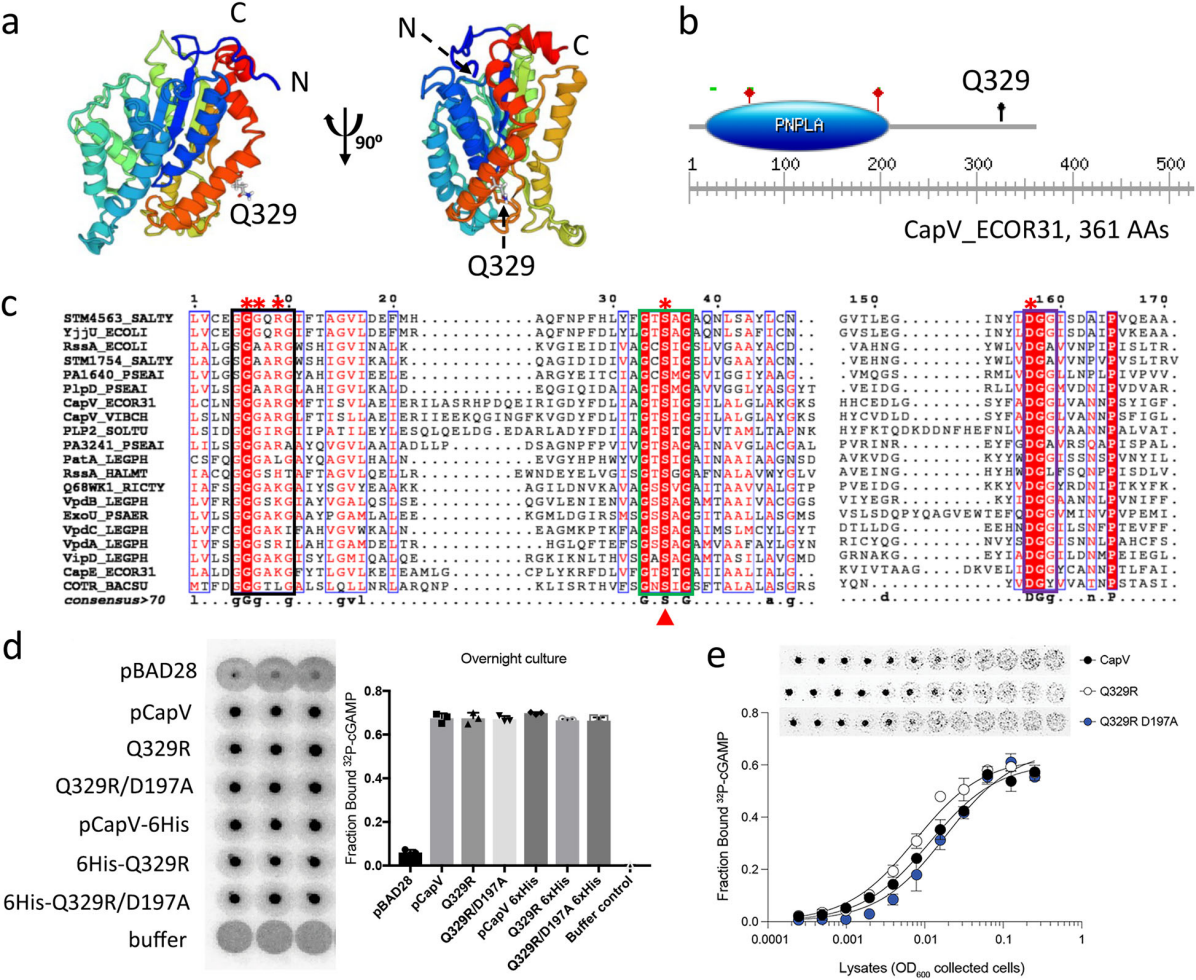
Bioinformatic analysis of the patatin-like phospholipase CapV of *E. coli* ECOR31 and capacity to bind cyclic AMP-GMP. **a** Predicted structural model of the CapV from *E. coli* ECOR31 shown as ribbon representation. The structural model was built with the I-TASSER server, the result was processed with SWISS-MODEL. The model was based on the coordinates of the 22% identical protein FabD from *Solanum cardiophyllum* (PDB: 1oxwC). **b** The graphical representation and schematic indication of the positions of the conserved motifs (indicated by the green bar) and putative active site residues S64 and D197 (marked by red stars) in the PNPLA domain of the 361 aa CapV from *E. coli* ECOR31 (from L19 to F210). Black arrow, Q329. The graph was assessed by ExPASy_Prosite. **c** Sequence alignment of CapV from *E. coli* ECOR31 and selected known phospholipases from other species establishes the conserved motifs of the PNPLA domain, G–G-G-x-[K/R]-G, G-x-S-x-G, and D–G-[A/G], boxed in black, green, and purple, respectively. Entirely conserved residues are shown in white on a red background. Conserved residues are boxed. Putative catalytic residues of CapV are indicated with filled red triangles. The residues in CapV_Q329R_ mutated to alanine are marked with red asterisks above the sequence. The consensus sequence at the bottom indicates in uppercase letter residues with 100% identity and in lowercase letter residues with higher than 70% conservation. Alignment was performed using CLUSTALW, and the result was processed with ESPript 3.0. Sequence identity as in the Methods section. **d** ^32^P-cAMP-GMP-DRaCALA of *E. coli* cell lysates expressing CapV, CapV_Q329R_, and CapV_Q329R/D197A_. VC = pBAD28; pCapV = CapV cloned in pBAD28; Q329R = CapV_Q329R_ cloned in pBAD28. D197A = CapV_Q329R/D197A_ cloned in pBAD28. **e** Assessment of affinity for cAMP-GMP of CapV, CapV_Q329R_, and CapV_Q329R/D197A_ expressed in *E. coli* MG1655. ^32^P-cAMP-GMP was mixed with twofold dilutions of cell extracts starting at a fourfold dilution.

Blast search with CapV from *E. coli* ECOR31 showed that CapV homologs with >60% identity are not only found in individual *E. coli* and *V. cholerae* strains, but are widely distributed among gamma-proteobacteria, including *Yersinia*, *Salmonella*, *Pseudomonas*, *Shewanella,* and *Klebsiella* species (Supplementary Fig. 4a). Phylogenetic analysis of representative CapV homologs supported classification into four different subgroups (Supplementary Fig. 4b). Alignment of the amino acid sequences of those CapV homologs showed that Q329 is a nearly invariant amino acid even among distantly related CapV proteins (Supplementary Fig. 4a). Q329 is, though, not required for catalytic activity and not part of other characteristic patatin-like phospholipase A2 (PNPLA) consensus motifs and the PNPLA core domain which is restricted to aa 19–210 (Prosite) in the 361 aa long protein. In order to clarify the position of R329 in the protein, we generated a structural model of CapV using the closest structural homolog from the PDB database as a template, the lysophospholipase-like protein FabD from *Solanum cardiophyllum* (PDB: 1oxwC; Fig. 3a) which shows 22% amino acid identity with CapV. According to this model, R329 is located within helix 12, the second last α helix of CapV_Q329R_ in the context of the RARGRR_329_ sequence pointing outward with no obvious change in the overall structure of the monomer or potential oligomer assembly to be observed.

As CapV from *V. cholerae*^46^, CapV of *E. coli* ECOR31 contains a N-terminal canonical PNPLA domain with three main characteristic conserved signature motifs^48,51^, the phosphate or anion binding motif G–G-G-x-[K/R]-G, the esterase box G-x-S-x-G, and the D–G-[A/G] motif as part of the catalytic dyad (Fig. 3b, c). The G-x-S-x-G motif includes the conserved nucleophilic serine 64 of the active site characteristic for the phospholipase A_2_ (PLA_2_) superfamily^48,52^.

To investigate if catalysis is required for swimming inhibition and filamentation upon CapV_Q329R_ overexpression, a catalytically inactive S64A variant of the protein (CapV_Q329R/S64A_) was generated. Compared with CapV_Q329R_, overexpression of CapV_Q329R/S64A_ equally inhibited swimming motility and induced filamentation (Figs. 1a and 2d, e), demonstrating that the G-x-S-x-G motif of CapV_Q329R_ is not required for the phenotype. However, the substitution of arginine in the G–G-G-x-[K/R]-G motif (CapV_Q329R/R27A_) and aspartic acid of the D–G-[A/G] motif (CapV_Q329R/D197A_) by alanine relieved both the repression of swimming motility and induction of filamentation. Substitution of the two structural glycine residues (CapV_Q329R/G24A/G25A_) of the G–G-G-x-[K/R]-G also partially suppressed swimming motility and induced only a mild filamentous phenotype upon overexpression of the protein (Figs. 1a, b and 2d, e). In summary, the G–G-G-x-[K/R]-G and D–G-[A/G] motifs of CapV_Q329R_ are required for repression of the swimming phenotype and cell filamentation.

As substitution of the nucleophilic serine 64 did not relieve motility and cell filamentation, we were wondering whether alternative serine residues are involved in the physiological activity of CapV_Q329R_. To this end, we substituted S33, S113/114, S146, S177, and S206 by alanine residues. Most of these serine residues were selected as they are located close to the catalytic site (Supplementary Fig. 2f). Only serine 206 was required to induce motility inhibition and promote cell filamentation by CapV_Q329R_ (Supplementary Fig. 2f).

Furthermore, we wanted to clarify whether specifically the Q329R mutation is required to induce filamentation. Replacement of Q329 by the other positively charged amino acid lysine still partially repressed the apparent swimming motility and induced filamentation (Fig. 1b), while replacement of Q329 by asparagine retained the wild-type CapV phenotype (Supplementary Fig. 2f, g).

The binding site for cAMP-GMP in CapV has not been identified. The Q329R substitution might alter the binding of cAMP-GMP or other cyclic dinucleotides to CapV. To this end, binding of cAMP-GMP to CapV and CapV_Q329R_ was analyzed by the differential radial capillary action of ligand (DRaCALA) assay. DRaCALA is based on the retention of small molecular compounds at the protein application spot on a nitrocellulose membrane upon binding. The experiment showed that both proteins bound cAMP-GMP with approximately equal affinity while the CapV_Q329R/D197A_ mutant showed diminished binding (Fig. 3d).

**Fig. 4:**
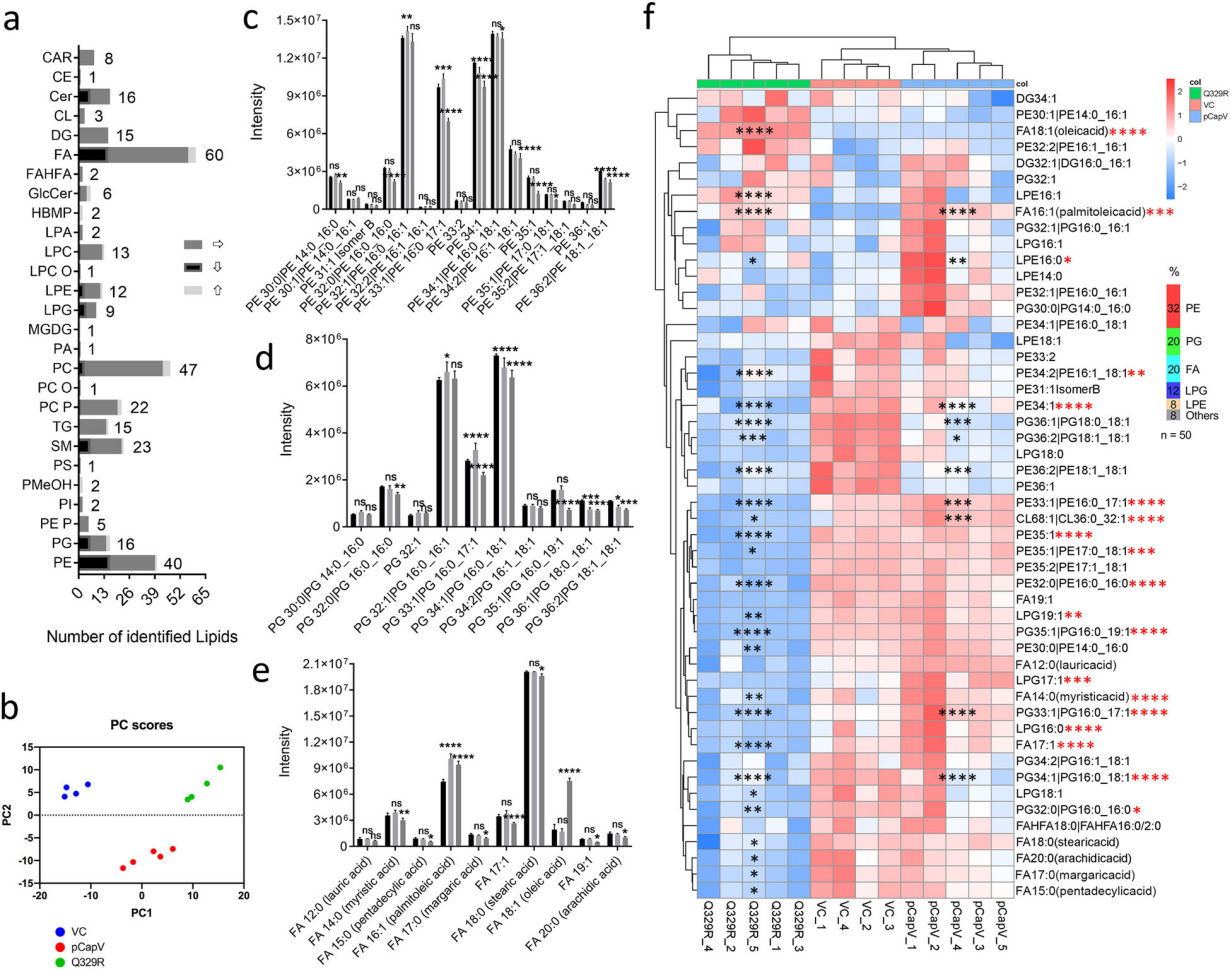
CapV and CapV_Q329R_ overexpression alter the steady-state lipid profile of *E. coli* MG1655. **a** The number of identified lipid species in CapV and CapV_Q329R_ induced filamentous cells compared to *E. coli* MG1655 vector control (VC) by untargeted charged surface hybrid column-quadrupole time-of-flight mass spectrometry (CSH-QTOF MS) analysis. For abbreviation of lipid compounds consult “Methods”. **b** Principle component analysis of lipid abundance upon overexpression of wild-type CapV and CapV_Q329R_ variant proteins in *E. coli* MG1655 compared to VC. Of six samples each, outliers have been removed. **c**–**e** Alternation and relative abundance of PE (**c**), PG (**d**), and FA (**e**) derivatives by untargeted CSH-QTOF MS analysis. Bars represent mean values from five independent replicates with error bars to represent SD. **f** Heatmap of selected 50 most significantly altered lipid species built based on hierarchical clustering. Each square represents one sample of each group. The color scale presenting the difference of each log_2_ transformed peak intensity value to the log_2_ transformed mean for each lipid species and the percentage of each lipid class is indicated on the right of the heatmap. Heatmap analysis was performed on the Tutools platform (https://www.cloudtutu.com), a free online data analysis website. Differences between mean values were assessed by two-tailed Student’s *t* test: ns, not significant; *P < 0.05, **P < 0.01, ***P < 0.001 and ****P < 0.0001; black stars in **c**, **d**, **e** and **f**: compared to MG1655 VC; red stars in **f**: statistical significance between *E. coli* MG1655 pCapV and *E. coli* MG1655 pCapV_Q329R_. VC = pBAD28. pCapV = CapV cloned in pBAD28; Q329R = CapV_Q329R_ cloned in pBAD28.

Patatin-like phospholipases hydrolyze the *sn*-2 acyl ester bond of neutral and phospholipids^48,53^. In order to assess whether overexpression of CapV and CapV_Q329R_ caused significant changes in the lipid profile concomitant with filamentation, we extracted lipids after 4 h of growth extracts to mass spectrometry (Fig. 4). Based on untargeted charged surface hybrid column quadrupole time-of-flight mass spectrometry (CSH-QTOF MS) analysis a total of 326 lipid species were identified (Fig. 4a). Principle component analysis showed distinct classification of samples into groups correlating with the overexpression of the wild-type CapV and CapV_Q329R_ variant protein (Fig. 4b), indicating that the lipid profiles are significantly altered. Subsequently, we applied hierarchal clustering analysis to segregate the samples cumulatively according to overall changes in the individual lipid compounds. Based on the changes in the lipid compounds, the samples can again be classified into distinct groups according to the expressed proteins (Fig. 4f). Lipids known to be most abundant in the *E. coli* membrane, phosphatidylethanolamines (PE), phosphatidyl-glycerols (PG) and fatty acids (FAs), displayed the highest relative peak intensity (Fig. 4c–e and Supplementary Fig. 5). We observed significant changes in the peak intensity of members of phospholipid classes, most abundant membrane components of *E. coli* such as PEs and PGs, but also of free FAs, lysophospholipids (LPE and LPG), phosphatidylcholines (PCs), ceramides (Cer) and sphingomyelins (SM), although the peak intensity of the latter three classes was at least 100-fold lower (Fig. 4c–e and Supplementary Fig. 5). The peak intensity was, however, in none of the cases on the average more than sixfold different. Notably, among the top 50 most significantly altered lipids, PE and PG derivatives with distinct FAs profiles are predominantly represented (Fig. 4f). While CapV overexpression showed downregulation of a restricted group of lipids, a significantly higher number of lipids species were downregulated upon CapV_Q329R_ overexpression. Notably, lipids species were also upregulated upon overexpression of CapV and CapV_Q329R_ suggesting a role of the patatin-like phospholipases in membrane reorganization and/or signaling. Only a few lipid species were distinctively upregulated upon overexpression of CapV_Q329R_, PE32:2 (16:1 16:1) with two monounsaturated fatty acids and the monounsaturated fatty acid FA18:1. Thus CapV and CapV_Q329R_ might have unique substrate profiles. Whether the observed alterations in the lipid profile are based on distinct residual catalytic activities of the two proteins or indirectly associated with the expression of CapV and CapV_Q329R_ needs to be investigated further. In summary, these results indicate that amino acids in the catalytic motifs are required to induce filamentation and to repress motility. Not or only partially activated CapV and CapV_Q329R_ can alter the lipid profile indicating that CapV is not only a receptor for a second messenger molecule, but might also be involved in alternative second messenger signaling in its nonactivated state.

### CapV_Q329R_ induces asymmetrically positioned FtsZ rings and abnormal nucleoids in filamentous cells

**Fig. 5:**
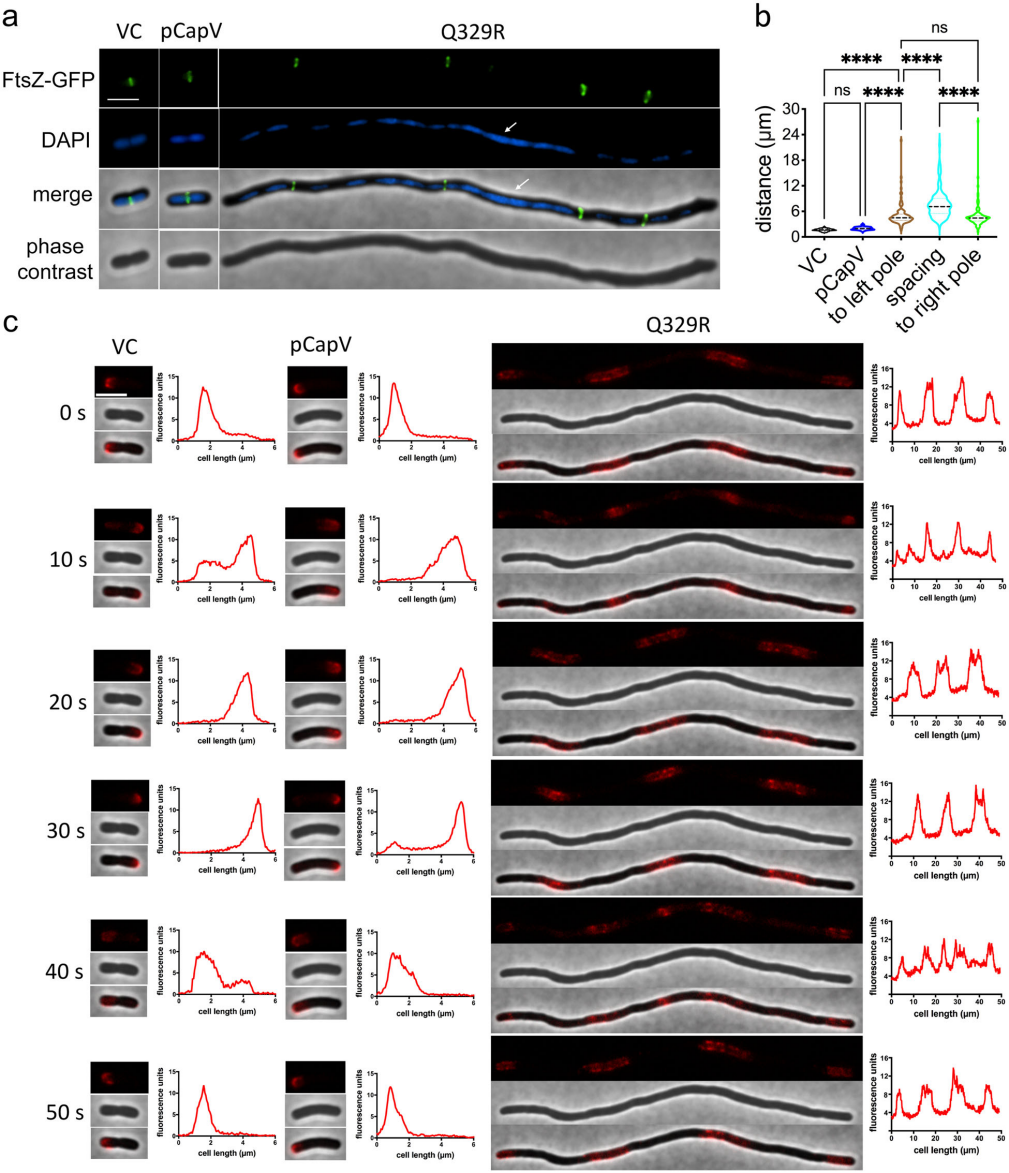
Cell division and chromosomal segregation, but not FtsZ-ring formation, is impaired in filamentous cells. **a** Phase-contrast and fluorescence images of FtsZ-GFP expressing cells (*E. coli* MG1655 derivative BS001 harboring vector control (VC), pCapV and pCapV_Q329R_). Cells were cultured in TB medium at 37 °C for 4 h, stained with DAPI, and assessed immediately under fluorescence microscopy. Large fragments of unsegregated nucleoids are indicated by white arrows. Bar, 3 µm. **b** Quantification of the average distance between two adjacent FtsZ rings upon overexpression of CapV and CapV_Q329R_, refers to Table 1. **c** Time-lapse analysis of mCherry-MinC expressing cells (*E. coli* MG1655 derivative PB318 harboring VC, pCapV and pCapV_Q329R_). A representative elongated cell is displayed. Graphs on the right of fluorescence images display the line profiles of fluorescent signals emanating from the cell. Arbitrary fluorescent units are obtained, analyzed by the Fiji ImageJ 1.8.0 software, and are plotted on the y axis; cell length (in µm) is plotted on the *x* axis. Bar, 3 µm. VC = pBAD28. pCapV = CapV cloned in pBAD28; Q329R = CapV_Q329R_ cloned in pBAD28.

During cell division, positioning of the FtsZ cytokinetic ring at the site of constriction between nucleoids is coordinated with chromosome replication, nucleoid segregation and cell elongation^31,54,55^. Impairment of this process leads to cell division arrest and filamentation, which can be induced by DNA damage and nucleoid occlusion^15,27^. After completion of cell segregation, the nucleoid subsequently becomes more compact^56^. To determine the effect of CapV and CapV_Q329R_ overexpression during the cell division process, we analyzed FtsZ-ring positioning in an *E. coli* K-12 MG1655 derivative with a chromosomally encoded FtsZ-GFP fusion protein^57^ and the position and shape of the nucleoid with DAPI staining. After induction of CapV and CapV_Q329R_ in liquid medium at 37 °C for 4 h, we immediately subjected the cells to fluorescence microscopy on agarose pads to visualize cell shape, septum, and FtsZ-ring formation and nucleoid location. Overexpressing wild-type CapV in the *E. coli* K-12 MG1655 FtsZ-GFP strain, we observed clearly visible constrictions that corresponded with a correctly positioned FtsZ ring and a single nucleoid in nondividing cells and two fully replicated and/or segregated nucleoids in dividing cells, respectively (Fig. 5a). Of note, nucleoids upon CapV expression appeared slightly more compact than those in the control.

In contrast, upon CapV_Q329R_-induced cell elongation, most filamentous cells displayed smooth contours with no visible septa (Fig. 5a and Supplementary Fig. 6a, b), suggesting that the block in cell division occurs prior to the development of constriction. However, one or two constrictions were occasionally observed in a few filaments (Supplementary Fig. 6a). We found that most of the CapV_Q329R_-induced filamentous cells were polynuclear and contained multiple abnormally shaped or positioned nucleoids. Discrete patches of DNA staining could be compact, asymmetrically positioned in the filament or displayed an extended or decondensed shape occupying an extensive part of the filament (Fig. 5a and Supplementary Fig. 6b). We also observed a few filamentous cells containing nucleoids, which were evenly positioned throughout the filament with larger interchromosomal spaces (Supplementary Fig. 6b). Collectively, these observations suggest filamentous cells induced by CapV_Q329R_ overexpression to be (partially) defective in chromosome segregation and nucleoid condensation.

Distinct FtsZ rings were observed in most of the filaments suggesting that the block in cell division occurred after FtsZ-ring formation. Large variations in the distance between two FtsZ rings lead to an unequal number of FtsZ rings in filamentous cells of similar length. CapV_Q329R_ promoted filamentation with an average cell length of 31.2 µm (*n* = 102), which corresponds to >15 times the standard cell length. The majority of the filamentous cells contained three FtsZ rings with multiple segregated and/or unsegregated nucleoids distributed between the rings. Of note, the distance between two adjacent Z-rings varied dramatically in the population of filamentous cells, with on average fourfold longer spacing in CapV_Q329R_ expressing cells compared to cells expressing CapV and the control (Fig. 5b and Table 1). Equally variable was the number of partitioned nucleoids among filamentous cells.

**Table 1:** Localization of FtsZ-GFP upon CapV_Q329R_ overexpression.

MG1655 harboring	No. of cells scored	Average cell length (μm)	% cells with the indicated no. of rings						Total no. of rings	Spacing of rings (μm/ring)^a^
			0	1	2	3	4	≧5		
pBAD28 vector	140	2.02	5	95	0	0	0	0	133	2.1
pCapV	140	2.39	3.6	96.4	0	0	0	0	135	2.5
pCapV_Q329R_	102	31.2	0	1	7.8	46.1	15.7	29.4	379	8.4

^a^Total length (column 2 multiplied by column 3) divided by total number of rings (column 4). The spacing is inversely proportional to the frequency of rings.

Abnormal FtsZ expression levels induce cell filamentation^58^. Comparative immunoblot analysis observed slightly lower FtsZ protein production levels in CapV_Q329R_ and CapV overexpressing cells, while the *ftsZ* mRNA steady-state level was with 91 and 97% of vector control expression only slightly changed (Supplementary Fig. 6c). Whether this (partial) decrease in FtsZ production levels is responsible for CapV_Q329R_-induced filamentation needs to be further investigated. In summary, CapV_Q329R_ inhibits cell division at the level of constriction initiation and interferes with nucleoid segregation and condensation.

### CapV_Q329R_-induced cell filamentation is *sulA*-independent

Previous studies showed that SulA blocks cell division by direct interaction with cell division core component FtsZ during the bacterial SOS response^19,32^. To investigate whether CapV_Q329R_ induced cell filamentation of *E. coli* MG1655 is caused by the activation of *sulA*, we analyzed cell morphology upon CapV_Q329R_ overexpression in a *sulA* mutant^59,60^. We found that CapV_Q329R_ induced the same filamentous cell phenotype in the *sulA* mutant as in wild-type *E. coli* MG1655 (Supplementary Fig. 6d), suggesting that CapV_Q329R_-induced cell filamentation is *sulA*-independent consistent with the observed FtsZ-ring formation.

### MinC oscillation takes place in filaments

Multiple factors coordinate the regulation of cell division^29^. Among them is MinC, which oscillates from pole to pole in order to prevent FtsZ-ring formation at the cellular poles^34^. Upon overexpression, MinC causes cell filamentation. We investigated the dynamics of MinC mobility by time-lapse fluorescence microscopy (Fig. 5c and Supplementary Movie 3). In rod-shaped wild-type cells, we observed that MinC oscillates between cell poles, moving a large fraction of the total fluorescence signals along the long axis of the cell as described previously^61^. A complete oscillation cycle lasted about 50 s under our experimental conditions. Of note, we found that oscillation of the FtsZ-ring inhibitor MinC is not affected in CapV_Q329R_-induced filaments (Fig. 5c) (PB318,^61^). MinC displayed various fluorescence spots ranging from one up to 5 depending on the length of the filament (Fig. 5c and Supplementary Fig. 6e). These fluorescent spots oscillated within multiple invisible cell borders in the filaments suggesting physical restrictions by septa. Within one single filamentous cell, the number of the apparent fluorescence fractions followed thereby a three-step principle: *n* *n* − 1 *n* (*n* ≧2, located at both poles) or *n* *n* + 1 *n* (*n* ≧1, located within the cell), during an oscillation periodicity of about 50 s, which is similar as for rod-shaped control cells and wild-type CapV overexpressing cells (Fig. 5c and Supplementary Fig. 6e). It will be relevant to investigate how long-distance MinC oscillation is maintained in filamentous *E. coli* cells. In summary, MinC oscillation might remain the effective watchdog for the prevention of FtsZ-ring formation in filamentous cells.

### Overexpression of CapV_Q329R_ reduces cell numbers

**Fig. 6:**
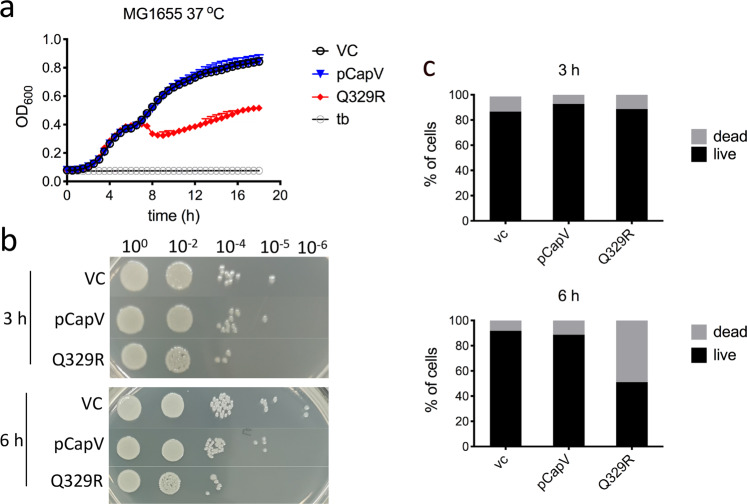
CapV_Q329R_, but not CapV is cytotoxic to *E. coli* MG1655 in the early stationary phase. **a** Growth curves of *E. coli* MG1655 upon CapV and CapV_Q329R_ overexpression induced by 0.1% l-arabinose in TB at 37 °C. Each data point represents the mean ± SD of six biological replicates. tb = TB medium. **b** Colony-spotting assay on agar plates. Cells were grown at 37 °C and harvested at different time points. Cell viability determined by spotting serial dilutions (10^0^–10^−6^) on LB plates to assess colony-forming units. **c** Quantification of Live/Dead staining of *E. coli* MG1655 cells after 3 h and 6 h in TB medium at 37 °C. *n* = 1200. VC = pBAD28. pCapV = CapV cloned in pBAD28; Q329R = CapV_Q329R_ cloned in pBAD28.

CapV has been identified as a patatin-like phospholipase that causes growth retardation upon activation by cAMP-GMP in *V. cholerae* El Tor^46^. In *E. coli* MG1655, though overexpression of CapV of *E. coli* ECOR31 wild-type did not affect cell division during the entire growth phase. Though no effect was observed during the first 3 h of cell growth in liquid culture, overexpression of CapV_Q329R_ induced a mild decrease in optical density after 6 h (Fig. 6a). As extensive filamentation might not permit a direct correlation between OD and cell number, we tested cell viability by spotting *E. coli* MG1655 cells on agar plates for counting of viable individual cells. Compared to the control and cells expressing CapV, ~50% of the colonies were recovered from *E. coli* MG1655 cell cultures expressing CapV_Q329R_ (Fig. 6b). Consistently, cells stained with the nucleic acid stain SYTO 9 for viability and propidium iodide (PI) for cell death showed that CapV_Q329R_ production induced approximately 50% *E. coli* MG1655 cells to stain selectively with PI after 6 h, while no DNA staining with PI was observed after 3 h (Fig. 6c and Supplementary Fig. 7a, b). Of note, cells in some filaments took up both dyes seemingly live and dead (Supplementary Fig. 7). Interestingly, the uptake of SYTO 9 into individual cells varied widely in particular in CapV expressing cells, which showed no decrease in cell viability. These data indicate a highly heterogeneous nucleic acid content in individual cells. Thus, expression of not or only partially activated CapV and CapV_Q329R_ might differentially affect membrane permeability and cell viability.

### Vitamin B6 restricts cell filamentation induced by CapV_Q329R_

**Fig. 7:**
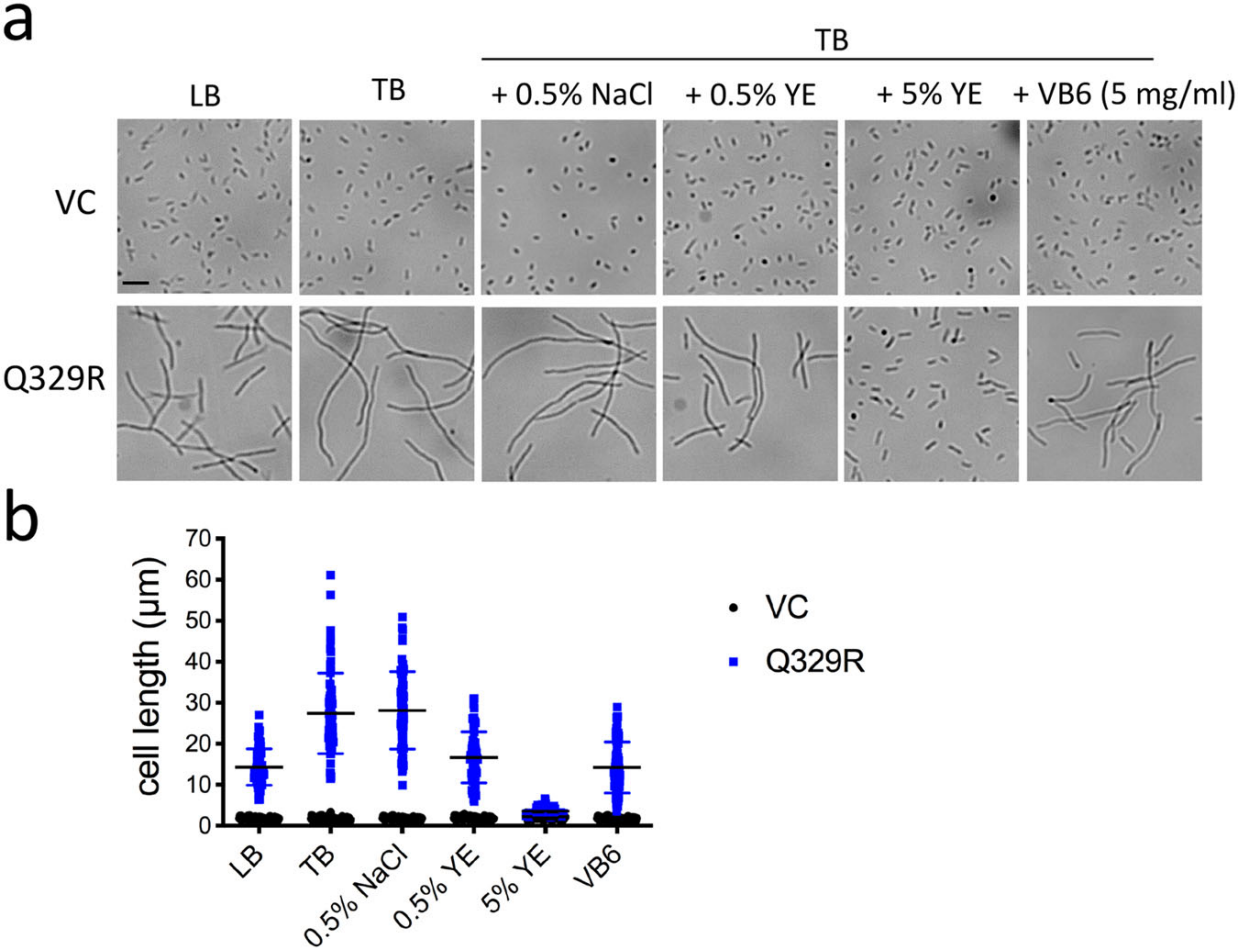
Vitamin B6 (pyridoxine) restricts cell filamentation of *E. coli* MG1655 induced by CapV_Q329R_. **a** Light microscopy pictures of *E. coli* MG1655 cell morphology and, **b** quantification of cell length in LB and TB medium supplemented with 0.5% NaCl, 0.5% YE, 5% YE, and VB6 (pyridoxine, 5 mg/ml), respectively. The quantification is based on results from at least three independent experiments with the assessment of 70 cells from each group. Bar, 5 µm. VC = pBAD28. Q329R = CapV_Q329R_ cloned in pBAD28.

Filamentation has been shown to be affected by environmental conditions^26^. We found that filamentation was particularly pronounced during cell growth in TB medium (1% tryptone, 0.5% NaCl), while it was restricted when cultured in LB medium (1% tryptone, 1% NaCl, 0.5% yeast extract) (Fig. 7a). Supplementation of TB medium with 0.5% yeast extract (YE) repressed the filamentous phenotype dramatically, while 5% restored the rod shape of all cells. Supplementation with 0.5% NaCl did not affect filamentation.

Yeast extract is the water-soluble portion of autolyzed yeast cells used to prepare microbiological culture media^62^. As a nutrient source, it provides nitrogen, amino acids, peptides, carbohydrates, vitamin B complex, and other components that promote microbial growth^63^. In particular, yeast extract contains B vitamins, water-soluble precursors of structurally unrelated enzyme cofactors including thiamine (B1), riboflavin (B2), nicotinamide (B3), pantothenate (B5), pyridoxine (B6), biotin (B7), folic acid (B9), and cobalamin (B12). To identify the component(s) in YE that contribute(s) to the repression of cell filamentation upon CapV_Q329R_ overexpression, we supplemented TB medium with these individual B vitamins. While supplementation with thiamine, riboflavin, nicotinamide, pantothenate, biotin, folic acid, and cobalamin showed no effect, supplementation with the vitamin B6 precursor pyridoxine at 5 mg/ml decreased the length of filaments by 50% (Fig. 7a, b and Supplementary Fig. 8). It will be relevant to investigate the molecular mechanism of pyridoxine to inhibit CapV_Q329R_-induced cell filamentation. Further, the functionality of interconvertible pyridoxal and pyridoxamine vitamin B6 complex compounds and their 5′ phosphate biologically active counterparts can be investigated individually and in combination. In summary, pyridoxine is one component in LB medium, which is effectively inhibiting cell filamentation upon overexpression of CapV_Q329R_.

### The effect of CapV_Q329R_ extends to commensal and UPEC *E. coli* strains and *S*. *typhimurium*

To determine if the effect of CapV_Q329R_ is restricted to *E. coli* K-12 MG1655, we overexpressed CapV_Q329R_ in commensal and UPEC *E. coli* strains^64,65^ and the gastrointestinal pathogen *S*. *typhimurium* UMR1. The NCBI database (accessed latest December 15, 2021) indicates CapV homologs to be encoded predominantly by human fecal *E. coli* strains and strains derived from animals and animal and plant products. UPEC *E. coli* can develop extended filamentation upon host cell escape^20^. In all cases, CapV_Q329R_ overexpression inhibited apparent swimming motility (Supplementary Fig. 9a, b).

Furthermore, CapV_Q329R_ induced different degrees of cell filamentation in the *E. coli* strains and *S*. *typhimurium* UMR1 (Supplementary Fig. 9c). CapV_Q329R_ production induced mild cell filamentation in commensal *E. coli* Fec32 and Fec89, moderate cell filamentation was observed in commensal Fec67 and extensive filamentation occurred in ECOR31, UPEC CFT073, and *S*. *typhimurium* UMR1. A heterogeneous population of moderately filamented cells as well as individual rod-shaped cells was observed in the UPEC strain *E. coli* No. 12 and the commensal *E. coli* strain Tob1 upon CapV_Q329R_ overexpression (Supplementary Fig. 9c). In summary, our results showed that the phenotypes observed upon CapV_Q329R_ expression were not restricted to *E. coli* MG1655, but were common to genetically unrelated commensal and UPEC *E. coli* strains and *S. typhimurium*, indicating a general effect of CapV_Q329R_ on bacterial cell morphology, regulation of flagella-mediated swimming motility and biofilm formation.

### Production of CapV_Q329R_ affects rdar biofilm formation on agar plates

We were subsequently wondering, whether CapV_Q329R_ affects phenotypes other than cell filamentation and flagella expression. To this end, we investigated the colony morphotype on agar plates. A significant number of *E. coli* isolates display a rdar biofilm morphotype on Congo red agar plates characterized by the expression of the extracellular matrix components amyloid curli and the exopolysaccharide cellulose^64^.

**Fig. 8:**
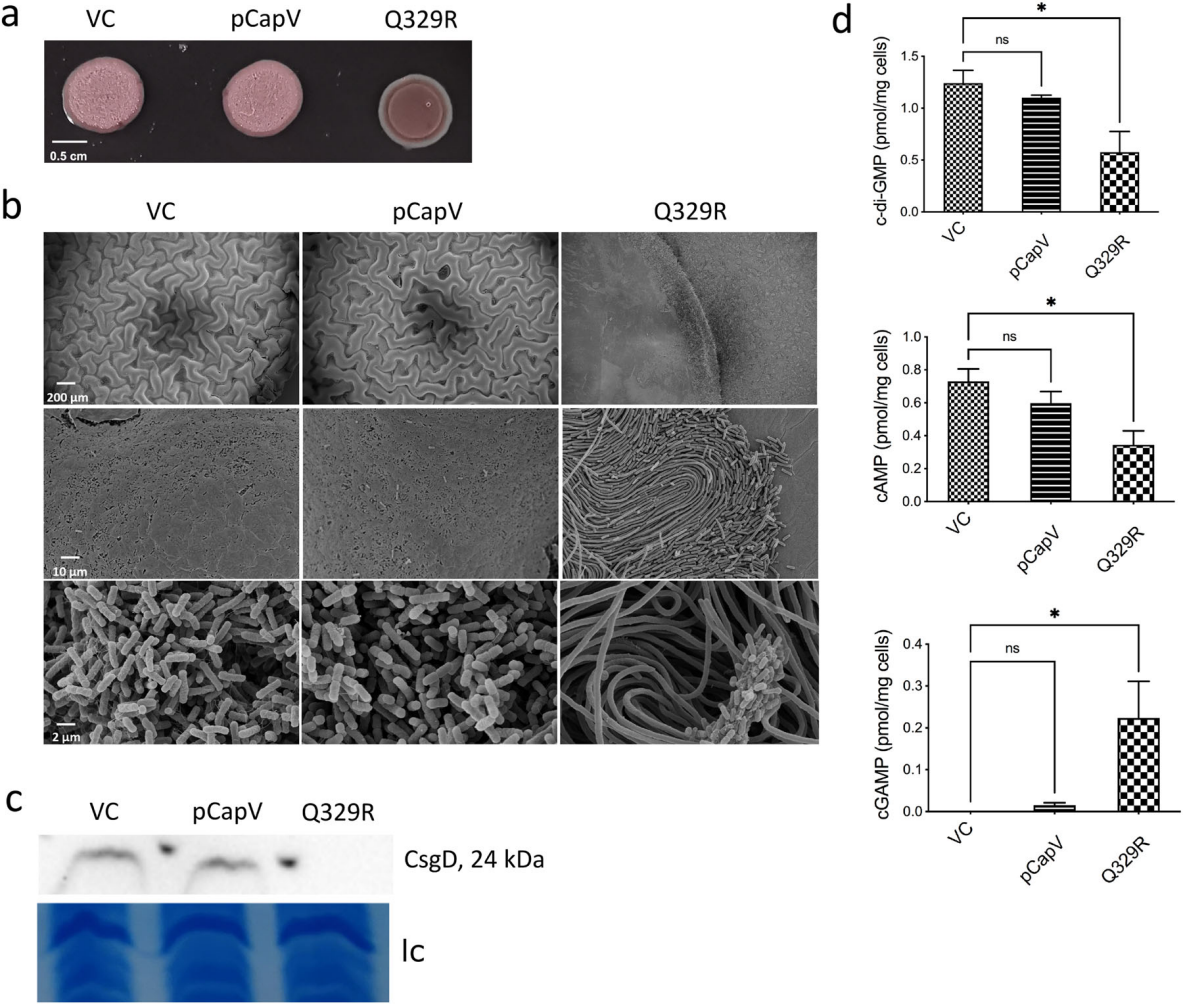
CapV_Q329R_ expression modulates rdar biofilm formation, CsgD expression, and cyclic (di)nucleotides levels in *E. coli* strain No. 12. **a** Rdar morphotype in wild-type *E. coli* No. 12 vector control (VC) and upon overexpression of CapV and its mutant CapV_Q329R_. Cells were grown on a salt-free LB agar plate for 24 h at 37 °C. **b** Scanning electron microscopy of plate-grown colonies. Colony morphotypes grown on a salt-free LB agar plate were fixed after 24 h of growth at 37 °C. **c** CsgD production upon overexpression of CapV and CapV_Q329R_ in *E. coli* strain No. 12 compared to VC. Only colony morphotypes from the same plate and signals from the same western blot are compared. **d** LC-MS/MS quantification of in vivo amounts of c-di-GMP, cAMP, and cAMP-GMP upon overexpression of CapV and CapV_Q329R_ in *E. coli* strain No. 12 compared to VC. Data are displayed as absolute amounts referred to the original cell suspension. VC = pBAD28. pCapV = CapV cloned in pBAD28; Q329R = CapV_Q329R_ cloned in pBAD28.

Congruent with the pronounced temperature-dependent effect of CapV_Q329R_, we investigated the effect of CapV_Q329R_ in strains that displayed the rdar morphotype at 37 °C. We choose the UPEC strain No. 12, which expresses a semi-constitutive *csgD*-dependent rdar morphotype^65^. While overexpression of wild-type CapV had no major effect compared to the No. 12 control, overexpression of CapV_Q329R_ dramatically disrupted the rdar morphotype (Fig. 8a). Observations of colonies by scanning electron microscopy and TEM demonstrated that CapV_Q329R_ induced even more extensive cell filamentation on agar plates compared to liquid culture, without affecting cell arrangement (Fig. 8b). While nonfilamented cells produced a pronounced extracellular matrix, the filaments produced only little or no matrix (Fig. 8b).

In conjunction with rdar morphotype downregulation, its major transcriptional activator CsgD was downregulated (Fig. 8c), demonstrating that CapV_Q329R_ expression affects the central regulatory hub for rdar biofilm formation in strain No. 12. Furthermore, CapV_Q329R_ overexpression equally downregulated the rdar morphotype in the commensal *E. coli* strains ECOR31, Fec67, Fec89, and Tob1 (Supplementary Fig. 9d). Downregulation of CsgD expression upon expression of CapV_Q329R_ was exemplarily observed for strain ECOR31 (Supplementary Fig. 9e).

We then investigated the effect of CapV_Q329R_ overexpression in commensal *E. coli* MG1655 and UPEC CFT073 which display a smooth and white morphotype when grown at 37 °C, indicative for the lack of rdar biofilm expression under these experimental conditions. After 48 h of growth, though, a distinctly structured brown colony morphology with dye uptake was displayed upon CapV_Q329R_, but not upon CapV overexpression (Supplementary Fig. 9d). In the same line, *S*. *typhimurium* strain UMR1 displayed a smooth and white morphotype at 37 °C^66^, with its colony to develop a similar brown morphotype after 48 h of CapV_Q329R_ overexpression (Supplementary Fig. 9d). Again, this colony morphotype seem to be distinct from the rdar biofilm in coloration with no or below detection limit production of the biofilm regulator CsgD in *E. coli* MG1655 (Supplementary Fig. 9). Semi-constitutive rdar morphotype expressing ECOR31 and No. 12 displaying reduced morphology and coloration retained low CsgD levels upon CapV_Q329R_ production (Fig. 8c and Supplementary Fig. 9c). Thus, CapV_Q329R_ production affects biofilm-associated colony morphotype formation in a complex way in different *E. coli* strains.

### CapV _Q329R_ expression alters cyclic (di) nucleotide concentrations

In *Enterobacteriaceae*, cyclic di-GMP is a ubiquitous bacterial second messenger, which stimulates the development of the rdar biofilm morphotype via *csgD* expression^40,67,68^. We investigated the cyclic (di) nucleotide levels exemplarily in the UPEC strain *E. coli* No. 12. Along with inhibition of *csgD* expression, the in vivo cyclic di-GMP level was concomitantly decreased upon CapV_Q329R_ overexpression (Fig. 8d). Besides cyclic di-GMP, both cAMP and cAMP-GMP regulate *E. coli* biofilm formation^43,69^. Consistent with a downregulated rdar biofilm phenotype, a reduction in cAMP and an increase in cAMP-GMP levels were observed upon CapV_Q329R_ overexpression (Fig. 8d). Thereby, CapV_Q329R_ might, for example, inhibit a diguanylate cyclase or promote phosphodiesterase activity to downregulate the cyclic di-GMP level. Taken together, these results indicate that CapV_Q329R_ inhibits rdar biofilm formation potentially through regulation of the intracellular level of various cyclic (di)-nucleotide signals.

### CapV_Q329R_ enhances the susceptibility of *E. coli* MG1655 to bacteriophage P1 infection and the antibiotic cephalexin

Recently, the patatin-like phospholipase CapV and the dinucleotide cyclase DncV have been shown to take part in bacterial antiphage defense^70^. Since CapV_Q329R_ showed a physiological function independent of DncV, we wondered whether CapV_Q329R_ still contributes to antiphage defense. While overexpression of wild-type CapV had no effect, interestingly, an approximately tenfold higher plaque formation was observed upon CapV_Q329R_ overexpression compared to the control when MG1655 cells were infected with P1 phage (Supplementary Fig. 10a), indicating that CapV_Q329R_ enhances susceptibility to bacteriophage P1 infection.

We also observed that overexpression of CapV_Q329R_, but not CapV renders the laboratory strain *E. coli* MG1655, the commensal strain ECOR31, and UPEC No. 12 more susceptible or sensitive against the beta-lactam antibiotic cephalexin (Supplementary Fig. 10b). A systematic investigation of altered susceptibility to various antibiotic classes awaits to be performed.

We were also wondering whether CapV_Q329R_-induced cell elongation and concomitant physiological changes impair the interaction of *E. coli* with host cells. To this end, we exposed the bladder epithelial cell line T24 to UPEC *E. coli* strain No. 12 overexpressing CapV and CapV_Q329R_. CapV_Q329R_ expressing *E. coli* strain No. 12 associated significantly less with the T24 bladder epithelial cell line than the control and CapV expressing cells. Although a trend to lower induction of mRNA steady-state levels of genes coding for the pro-inflammatory markers IL-1β and CXCL-8 was observed for T24 cells exposed to CapV_Q329R_ expressing bacteria, the obtained results were not statistically significant (Supplementary Fig. 10c). Thus, in summary, CapV_Q329R_ production causes substantial physiological alterations in *E. coli* MG1655.

## Discussion

Rod-shaped bacteria can undergo filamentation upon dysregulation of cytokinesis components. In this study, we describe a variant of the patatin-like phospholipase CapV containing a single amino acid substitution, CapV_Q329R_, to repress the apparent swimming motility and to induce extensive *sulA*-independent cell filamentation in *E. coli* MG1655, while only slightly decreasing FtsZ levels. Besides these substantial morphological changes, which occur in both commensal and pathogenic *E. coli* strains, modulation of rdar biofilm formation occurs concomitantly in the stationary phase of growth. Alteration in local or overall concentrations of cyclic (di)-nucleotides upon CapV_Q329R_ overexpression might be involved in motility and rdar biofilm regulation. Collectively, these findings demonstrate a single amino acid change to create a protein, which dramatically modulates various aspects of bacterial physiology.

CapV of *E. coli* ECOR31 is a patatin-like PLA2 that contains a N-terminal PNPLA domain and a C-terminal domain of unknown function (Fig. 3b, c). Patatin-like phospholipases proteins are predominantly phospholipase A2 enzymes, acyl hydrolases cleaving the *sn*-2 position of neutral lipids and phospholipids. We did not observe fundamental alterations in the lipid profile after 4 h of CapV_Q329R_ induction where extensive filamentation had already occurred indicating that CapV and CapV_Q329R_ display only a minor, if any, catalytic activity in the genetic background of *E. coli* MG1655 (Fig. 3d, e, f and Supplementary Fig. 4). In plants, patatin-like phospholipases do not only act as enzymes to cleave fatty acids from membrane lipids but also aid in the control of the spread of infection. Such a protein is, for example, highly abundant as a storage protein in potato tubers^71,72^. In mammals, PLPs have been recognized to be involved in lipid metabolism and turnover^73^, with a polymorphism in a patatin-like phospholipase to provide the genetic predisposition for nonalcoholic fatty liver disease and metabolic syndrome^74^. Furthermore, a patatin-like phospholipase is involved in axon functionality of neurons^75^. Patatin-like phospholipases are also found in pathogenic bacterial species and act as toxins in host–pathogen interactions^53,76^. One of the best characterized patatin-like phosphlipases is ExoU, a cytotoxic effector protein of *P. aeruginosa* secreted through the type III secretion system upon host cell contact^77^. Interaction with host ubiquitin and ubiquitinated proteins triggers the PLA2 activity of ExoU leading to degradation of host cell membranes^78^. Our finding that the G–G-G-x-[K/R]-G and D–G-[A/G] motifs in the PNPLA domain of CapV_Q329R_, but not the catalytic G-x-S-x-G motif, are required for the cell filamentation phenotype suggests a functional role for the protein scaffold (Fig. 2d). Since bacterial filamentation also contributes to pathogenesis by escaping from phagocytosis, we hypothesize that CapV_Q329R_ has a role in bacterial survival upon induction of cell filamentation during interaction with host immune cells.

The amino acid Q329 is located C-terminal of the putative PNPLA domain of CapV of *E. coli* ECOR31 and consequently not part of the characteristic motifs required for the catalytic activity of this PLA2 superfamily member (Fig. 3b). 3D model construction using the closest homolog of CapV from *E. coli* ECOR31 in the PDB database (FabD from *Solanum cardiophyllum*, PDB: 1oxwC) revealed that Q329 is located in the context of the RARGRR_329_ sequence motif within the second last α helix of the CapV model structure with the arginine side chain pointing outwards, causing no change in the overall structure (Fig. 3c). However, the switch from a neutral hydrogen-bond acceptor (Q) to a hydrogen-bond donor (R) with a longer side chain indicates that CapV_Q329R_ might show altered enzymatic activity, ligand binding properties or protein-protein interactions. Arginine with its positively charged side chain can be involved in a variety of different functionalities such as in binding of negatively charged phosphates of DNA molecules or nucleotides. The RR twin-arginine motif is part of the N-terminal signal sequence for the Twin-Arginine Translocation (TAT) pathway^79,80^. Furthermore, a RxxR motif constitutes a conserved peptidase cleavage site, while a RxxxR motif is part of the binding motif for c-di-GMP in PilZ domain proteins, whereby the arginine residues bind O-6 and N-7 at the Hoogsteen edge of the guanine base^81^.

The catalytic activity of bacterial and eukaryotic patatin-like phospholipases is highly regulated to require a cofactor for activation^48^. CapV from *V. cholerae* El Tor is activated by binding to 3′3′-cAMP-GMP synthesized by the dinucleotide cyclase DncV to cause growth retardation^46^. Up to now, the binding site of cAMP-GMP in CapV awaits identification, while our preliminary experimentation suggests that CapV_Q329R_ and CapV bind cAMP-GMP with equal affinity. Our result showed that the CapV variant CapV_Q329R_ did hardly affect *E.* *coli* MG1655 cell viability in the logarithmic growth phase, but induced a 50% growth retardation and loss of cell viability in the stationary phase (Fig. 5 and Supplementary Fig. 7). We cannot exclude cAMP-GMP binding to be required for expression of motility and filamentation phenotypes, but consider this as unlikely. The in vivo cAMP-GMP concentration was below the detection limit for the *E. coli* K-12 MG1655 strain. Cyclic AMP-GMP could be produced by a GGDEF diguanylate cyclase with altered substrate specificity in the uropathogenic strain No. 12 upregulated upon CapV_Q329R_ expression. Alternatively, c-di-GMP binding could be required for residual stimulation of catalytic activity. As the catalytic serine residue in the G-x-S-x-G motif of the PNPLA domain is not involved in triggering motility inhibition and cell filamentation, we cannot exclude that CapV_Q329R_ might induce the respective phenotypes by residual enzymatic activity, solely through its altered protein scaffold (independent of cAMP-GMP binding) or modification of an enzymatic activity other than the phospholipase activity.

Bacterial cell filamentation is often associated with activation of the SOS response and the cell division inhibitor SulA^16,32^. However, also mutations in metabolic genes cause filamentation in *E. coli*^19^. According to our results, CapV_Q329R_ induced a *sulA*-independent filamentation in MG1655 (Supplementary Fig. 6d), indicating CapV_Q329R_ affects cell morphology possibly via an alternative signaling pathway.

Besides filamentation, CapV_Q329R_ overexpression inhibited apparent swimming motility of *E. coli* MG1655 in the soft agar plate (Fig. 1a), with CapV_Q329R_ overproducing cells concomitantly to gradually loose flagella to become non-motile (Supplementary Movie S1). Long filamentous cells, even if observed being motile when up to 20 times the length of a wild-type cell in liquid culture, might become physically trapped in the pores of the agar.

Interestingly, CapV_Q329R_ also downregulated rdar biofilm formation in several *E. coli* strains that express the morphotype at 37 °C (Supplementary Fig. 9d and Fig. 7a). Examination of cells showed CapV_Q329R_ to remodel colony morphology on the agar plate with the appearance of highly filamented cells along with a light-brown colony morphotype (Fig. 7b). Besides the reduction of in vivo c-di-GMP level, a reduction in cAMP level and a weak signal for cAMP-GMP were detected upon CapV_Q329R_ overexpression in the UPEC *E. coli* strain No. 12 (Fig. 7d). Cyclic AMP has been shown to promote extracellular matrix production and biofilm formation in UPEC^69^. Our previous investigation showed that DncV-synthesized cAMP-GMP participates in the downregulation of rdar biofilm formation in *E. coli* ECOR31^43^. Besides DncV, a certain class of GGDEF domain proteins has been identified to catalyze the synthesis of cAMP-GMP^82^. Inspection of the genome sequence did not indicate the presence of a *dncV* homolog in the *E. coli* No. 12 genome (unpublished observation), suggesting that cAMP-GMP production by a GGDEF domain protein(s) might be directly or indirectly activated by CapV_Q329R_, to inhibit rdar biofilm formation of *E. coli* No. 12. Furthermore, the induction of a *csgD* independent alternative colony morphotype with distinct dye-binding capability in isolates with a smooth and white morphotype points to a complex regulation of bacterial colony morphology by CapV_Q329R_.

We show in this work that CapV_Q329R_ expression increased susceptibility to infection by the myophage P1 (Supplementary Fig. 10a). Of note, gene products of the *capV-dncV-vc0180*-*vc0181* operon (Supplementary Fig. 1) of *E. coli* and *V. cholerae* in distinct combinations confer immunity against various bacteriophages^70^. Upon phage infection, the dinucleotide cyclase DncV catalyzes the synthesis of cAMP-GMP which in turn activates the patatin-like phospholipase CapV to eventually abort infection by leading to cell death^70^. As a mechanism of increased susceptibility, CapV_Q329R_ might alter the abundance of the terminal glucose on core lipopolysaccharide, which serves as the P1 receptor thereby increasing phage-bacterial interaction, enhance the efficiency of DNA injection or manipulate other mechanisms to lead to higher effectivity of P1 infection. Whether the effect of CapV_Q329R_ on phages infection extends to alternative phages needs to be investigated.

**Fig. 9:**
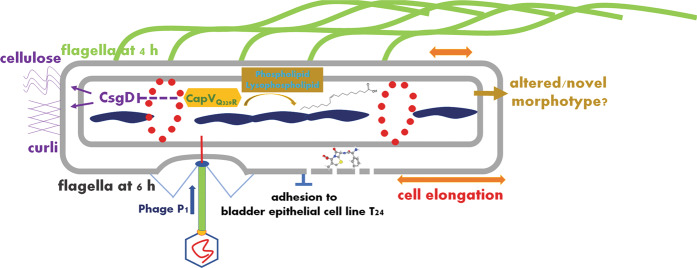
Summary of morphological and physiological changes caused by the production of the patatin-like phospholipase variant CapV_Q329R_ in *E. coli*. Overexpression of CapV_Q329R_ in *E. coli* MG1655 causes substantial morphological and physiological changes which lead to cell elongation with extensive filamentation as a consequence, disparate FtsZ-ring formation, impaired nucleoid segregation, and condensation. a change in the overall lipid profile of the cells, downregulation of flagella expression and motility, downregulation of rdar biofilm morphology and production of its activator CsgD and enhanced susceptibility to infection by the myophage P1 and the cell wall inhibiting antibiotic cephalexine. In the clinical isolate *E. coli* No. 12, reduced adhesion to the bladder epithelial cell line T24 has been observed. In strains not producing the rdar morphotype at 37 °C, a potentially novel biofilm morphotype has been observed. Cephalexin structure from PubChem (https://pubchem.ncbi.nlm.nih.gov/compound/Cephalexin); structure of oleic acid from Wikipedia (https://en.wikipedia.org/wiki/Oleic_acid).

In conclusion, our study reported CapV_Q329R_, a putative patatin-like phospholipase, to inhibit apparent swimming motility and rdar biofilm formation, while to promote extensive cell filamentation, antimicrobial susceptibility and susceptibility to phage P1 (Fig. 9). The major physiological effects extend beyond the laboratory model *E. coli* K-12 MG1655 to various commensal and clinical *E. coli* strains and *S. typhimurium*, demonstrating a single amino acid substitution to evolve protein functionality towards a dramatic modulation of various aspects of bacterial physiology. It remains to be shown whether CapV_Q329R_ can demonstrate physiological effects even beyond these two closely related enterobacteria and whether other phospholipid bilayer-modifying enzymes show a similar effect.

## Methods

### Bacterial strains and growth conditions

All strains used in this study are listed in Supplementary Table S1. Strains were cultured in tryptone broth (TB, 1% tryptone, 0.5% NaCl), Luria-Bertani (LB, 1% tryptone, 0.5% yeast extract, 1% NaCl) liquid medium or on LB agar plates supplemented with 25 µg/ml chloramphenicol (Cm) at indicated temperatures. l-arabinose (Sigma) was used for induction of gene expression at indicated concentrations.

### Plasmid construction

All plasmids and primers used in this study are listed in Supplementary Tables S1–S3, respectively. Genes of interest were amplified by PCR, the PCR products digested with indicated restriction enzymes and ligated into the pBAD28 vector using the Rapid DNA Ligation Kit (Roche Diagnostics). Inserted DNA sequences were confirmed by DNA sequencing (StarSeq).

### Site-directed mutagenesis

Site-directed mutagenesis was performed using the Q5 site-directed mutagenesis kit according to the manufacturer’s instructions (NEB). All mutations were confirmed by DNA sequencing.

### Swimming assay

To assess apparent swimming motility, 3 µl of an overnight culture suspended in water (OD_600_ = 5) was inoculated into soft agar medium containing 1% tryptone, 0.5% NaCl, and 0.25% agar^83^. The swimming halo was documented with a Gel Doc imaging system (Bio-rad) after 6 h at 37 °C and the swimming diameter was measured by ImageJ 1.8.0^84^.

### Rdar colony morphotype assessment

To visualize expression of cellulose and curli fimbriae, 5 µl of an overnight culture of OD_600_ = 5 suspended in water was spotted onto LB without NaCl agar plates containing the dye Congo red (40 µg/ml, Sigma) and Coomassie Brilliant Blue G-250 (20 µg/ml, Sigma). Plates were incubated at 37 °C. Pictures were taken at different time points to analyze the development of the colony morphology structure and dye binding.

### Antimicrobial susceptibility assay

Antimicrobial susceptibility was assessed by the disc diffusion assay. Cells were spread on a Mueller Hinton agar plate containing 0.1% arabinose and 25 µg/ml Cm. After 3 h of incubation at 37 °C, discs with 30 µg cephalexin were placed and incubation was continued for another 16 h. Zones of inhibition and sensitivity were assessed visually and by measuring the radius of the zone of inhibition and sensitivity as indicated by no and reduced growth, respectively.

### Isolation of cell-associated flagellin

Bacterial cell-associated flagellin was isolated as described previously.^85^ Briefly, a single bacterial colony of each group was inoculated in TB medium and cultured overnight at 37 °C at 150 rpm. After dilution to OD_600_ = 0.01, 0.1% l-arabinose was added for induction of the protein, and culturing was continued for indicated time points. Flagella were sheared off by forcing the sample 15 times through a syringe with a needle of 0.51 mm diameter (BD Microlance). One ml of samples was collected, centrifuged at 17,000 rpm and the supernatant was mixed with cold trichloroacetic acid (v:v = 3:1, Sigma). Samples were incubated at −20 °C for 2 h, followed by centrifugation at 17,000 rpm for 40 min at 4 °C. The cell pellet was collected for SDS-PAGE analysis (4% stacking gel, 12% running gel) and the gel was stained by colloidal Coomassie brilliant blue (Sigma). Uncropped gels are exemplarily shown in the Supplementary Information file.

### Flagella staining

Staining of bacterial flagella according to Leifson was performed using a flagella stain kit according to the instructions of the manufacturer (BASO Diagnostics). Flagella were observed under a light microscope (Leica MC170 HD).

### Transmission electron microscopy

Bacterial flagella were visualized by transmission electron microscopy (TEM)^43^. Briefly, a suspension of bacterial cells grown overnight was diluted in TB medium with protein expression induced by 0.1% l-arabinose for indicated time points. An aliquot of 3 µl from each sample was added to a grid with a glow discharged carbon-coated supporting film for 3 min. The excess solution was soaked off by a filter paper and the grid was rinsed by adding 5 µl distilled water for 10 s. Distilled water was soaked off again and the grid was stained with 5 µl 1% uranyl acetate (Sigma) in water for 7 sec. Excess stain was soaked off and the grid was air-dried. The samples were examined in a Hitachi HT 7700 (Hitachi, Tokyo, Japan) electron microscope at 80 kV and digital images were taken with a Veleta camera (Olympus, Münster, Germany).

### Scanning electron microscopy

The bacterial colonies were exposed to vapor from a filter paper soaked with 4% glutaraldehyde fixed onto the lid of the petri dish immediately after growth. Subsequently, colonies were fixed in 1.5 ml fixative solution (0.5% glutaraldehyde, 2.5% paraformaldehyde in 10 mM HEPES, pH 7.0) for 2 h at 4 °C. The fixed biofilm sample was processed for observation by scanning electron microscopy (SEM) applying dehydration with acetone, critical-point drying, and sputters coating with gold/palladium. Samples were examined in a Zeiss Merlin field emission scanning electron microscope at an acceleration voltage of 5 kV with the Everhart-Thornley SE-detector and the inlens SE-detector in a 30:70, 70:30, or 77:23 ratio.

### Light and fluorescence microscopy

Cell morphology was investigated either under the light microscope (Leica MC170 HD) or a fluorescence microscope (Nikon). The viability of cells was analyzed with the LIVE/DEAD BacLight fluorescence stain from Life Technologies (Thermo Fisher Scientific). For all measurements, live cells were mounted onto 1% agarose pads supplemented with TB medium prior to microscopy analysis. Phase-contrast and fluorescence images were captured using a Ti eclipse inverted research microscope (Nikon) with a ×100/1.45 numerical aperture (NA) objective (Nikon). Image processing and cell length measurement was conducted by Fiji ImageJ 1.8.0^84^.

### Lipid extraction

Lipid extraction was performed as described previously with slight modifications^46,86^. Briefly, overnight cultures were inoculated into 50 ml of TB to OD_600_ = 0.01, expression of gene products induced by 0.1% arabinose, and grown for 4 h at 37 °C. Cells were collected by centrifugation at 4 °C for 20 min, and 5 ml organic extraction buffer (methanol:chloroform:0.1 M formic acid = 20:10:1, v/v/v) was added to the cell pellets, followed by 1 h of heavy shaking at room temperature. 2.5 ml inorganic aqueous buffer (0.2 M H_3_PO_4_, 1 M KCl) was added, followed by another 1 h of heavy shaking. The mixture was centrifuged at 13,000×*g* for 10 min and lipids dissolved in the lower chloroform phase were collected for analysis by mass spectrometry.

### Lipidomics analysis

Extracted lipids were analyzed using ultra-high-pressure liquid chromatography (UHPLC) on a Waters CSH column, interfaced to a quadrupole/time-of-flight (QTOF) mass spectrometer (high resolution, accurate mass), with a 15 min total run time. The LC/QTOF MS analyses were performed using an Agilent 1290 Infinity LC system (G4220A binary pump, G4226A autosampler, and G1316C Column Thermostat) coupled to either an Agilent 6530 (positive ion mode) or an Agilent 6550 mass spectrometer equipped with an ion funnel (iFunnel; (negative ion mode). Lipids were separated on an Acquity UPLC CSH C18 column (100 × 2.1 mm; 1.7 µm) maintained at 65 °C at a flow rate of 0.6 ml/min. Solvent pre-heating (Agilent G1316) was used. The mobile phases consisted of 60:40 acetonitrile:water with 10 mM ammonium formate and 0.1% formic acid (A) and 90:10 propan-2-ol:acetonitrile with 10 mM ammonium formate and 0.1% formic acid. The gradient was as follows: 0 min 85% (A); 0–2 min 70% (A); 2–2.5 min 52% (A); 2.5–11 min 18% (A); 11–11.5 min 1% (A); 11.5–12 min 1% (A); 12–12.1 min 85% (A); 12.1–15 min 85% (A).

Samples were injected (1.7 µl in positive mode and 5 µl in negative ion mode) with a needle wash for 20 s (wash solvent is isopropanol). The valve is switched back and forth during the run for washing to reduce carryover of less polar lipids. The sample temperature is maintained at 4 °C in the autosampler.

Data from six samples for each experiment were collected in both positive and negative ion mode, and analyzed using MassHunter (Agilent). Lipids were identified based on their unique MS/MS fragmentation patterns using the software Lipidblast (https://fiehnlab.ucdavis.edu/projects/LipidBlast).^87^

Principle component analysis was performed on the datasets with GraphPad 9.0 software. The heatmap was constructed on the website https://www.cloudtutu.com/#/login using default parameters with average as the clustering method. In short, values for peak height were log_2_ transformed, the mean for each lipid species calculated and subsequently each value was mean centered and displayed as a color code.

Abbreviation of lipid compounds as follows: PE, phosphatidylethanolamine; PG, phosphatidylglycerol; PE P, plasmenyl-phosphatidylethanolamine; PI, phosphatidylinositol; PMeOH, phosphatidylmethanol; PS, phosphatidylserine; SM, sphingomyelin; TG, triglyceride; PC P, plasmenyl-phosphatidylcholine; PC O, plasmanyl-phosphatidylcholine; PC, phosphatidylcholine; PA, phosphatidic acid; MGDG, monogalactosyldiacylglycerol; LPG, lysophosphatidylglycerol; LPE, lysophosphatidylethanolamine; LPC O, plasmanyl-lysophosphatidylcholine; LPC, lysophosphatidylcholine; LPA, lysophosphatidic acid; HBMP, 1-Monoacylglycerol-phospho-2,3-diacylglycerol; GlcCer, glycosylceramide; FAHFA, fatty acid ester of hydroxyl fatty acid; FA, fatty acid; DG, diacylglycerol; CL, cardiolipin; Cer, ceramide; CE, cholesteryl ester; SM, sphingomyelin; CAR, acylcarnitine.

### Extraction of in vivo-produced nucleotides

Extraction of cyclic dinucleotides from bacterial cells was performed as reported^88^. Briefly, individual colonies were inoculated in TB medium, overnight cultures diluted to OD_600_ = 0.01, and grown in TB medium at 37 °C for 4 h containing 0.1% L-arabinose to induce expression of CapV and CapV_Q329R_. In all, the 5-ml cell suspension was pelleted and resuspended in 500 µl ice-cold extraction solvent (acetonitrile/methanol/water/formic acid = 2/2/1/0.02, v/v/v/v), followed by boiling for 10 min at 95 °C. Three subsequent extracts were combined and frozen at −20 °C overnight. The extracts were centrifuged for 10 min at 20,800×*g*, evaporated to dryness in a Speed-Vac (Savant), and analyzed by LC-MS/MS^89,90^. The detection limits for cyclic di-GMP and cAMP were 0.065 and 0.031 pmol/mg wet cell weight, respectively. The detection limits for cAMP-GMP and cyclic di-AMP were 0.235/0.027 and 0.054/0.012 pmol/mg for *E. coli* strains No. 12/MG1655, respectively.

### Western blot analysis

To detect protein expression, 5 mg (wet weight) of bacterial cells collected from agar plates or liquid cultures were resuspended in 200 µl SDS sample buffer and heated at 95 °C for 10 min. The total protein content was assessed by Coomassie Brilliant blue staining after sample separation. Samples containing equal amounts of protein were separated by SDS-PAGE (4% stacking and 12% resolving gel) and transferred onto a PVDF membrane (Millipore). The membrane was blocked with 5% skim milk (for detection of *E. coli* CsgD and flagellin FliC) or 5% BSA (for detection of His-tagged protein) in blocking buffer overnight. CsgD and FliC were detected with a polyclonal *E. coli* anti-CsgD peptide primary antibody (dilution 1:5000)^91^ and anti-Flagellin (FliC) antibody (ab93713, Abcam, dilution 1:5000), respectively. The horseradish peroxidase-conjugated goat anti-rabbit IgG was the secondary antibody (154-10004301, Jackson ImmunoResearch Laboratories Inc., dilution 1:2000). An anti·His antibody conjugated to horseradish peroxidase (Penta·His HRP Conjugate, ID: 34460, Qiagen, dilution 1:2000) was used to detect 6xHis-tagged proteins. Antibody binding was visualized with ECL light detection reagent (Roche) using Luminescent Image Analyzer (LAS-1000plus, Fujifilm). Uncropped blots are exemplarily shown in the Supplementary Information file.

### Total RNA isolation and quantitative real-time PCR (qPCR) of bacterial cells

Bacterial cells were grown in TB medium for 6 h with CapV/CapV_Q329R_ expression induced from the pBAD28 vector. Total RNA was isolated by hot acidic phenol extraction as previously described^92^ with subsequent assessment of RNA concentration by recording an absorbance spectrum from 220 to 350 nm by the NanoDrop 2000 (Thermo Scientific) and assessment of RNA integrity and DNA contamination upon separation in a 1% agarose gel run with 0.5 × TBE buffer. In total, 1 µg of RNA was used for cDNA synthesis by the High-Capacity cDNA reverse transcription kit (Applied Biosystems). Quantitative PCR was run in the ABI 7500 real-time PCR system (Applied Biosystems) using the RT-qPCR ReadyMix^TM^ (Sigma) with primers indicated in Supplementary Table S4. Reference genes were *recA* and *rspV*. The reference for relative target gene expression was the expression in the vector control strain calculated as 2^−∆∆CT^.

### Phage P1 plaque-formation assays

The phage P1 plaque-formation assay was performed according to previously described methods with slight modifications^70,93^. Individual bacterial colonies from overnight cultures were inoculated into TB medium and grown to OD_600_ = 0.1 at 37 °C, followed by further growth with 0.1% l-arabinose for another 3 h to induce the expression of CapV and CapV_Q329R_. For the determination of phage infectivity by plaque formation, 500 µl of cells were thoroughly mixed with 4.5 mL modified MMB agar (TB with 0.1 mM MnCl_2_, 5 mM MgCl_2_, 0.35% agar), immediately poured onto Petri dishes containing 20 ml MMB Agar (1.6%) and allowed to cool for 10 min at room temperature. Tenfold serial dilutions of P1 phage lysate in SM Buffer (100 mM NaCl, 8 mM MgSO_4_, 50 mM Tris-HCl pH 7.5) were dropped on top of the double-layer agar plate and allowed to dry for 20 min at room temperature. Plates were incubated at 37 °C for 18 h and plaques were counted to compare the efficiency of plating.

### Bioinformatic analyses

A BLAST search against the NCBI protein database was performed with standard parameters using the *E. coli* ECOR31 CapV sequence as a query. All distinct protein sequences with >40% identity from different species were selected. Sequences were aligned using CLUSTALW^94^ and processed with ESPript 3.0^95^ using standard parameters. The phylogenetic tree was reconstructed by calculating sequence similarity by Maximum Likelihood (ML) in MEGA 7.0^96^. A CapV and CapV_Q329R_ structural model was built with the I-TASSER server^97^ and processed with SWISS-MODEL^98^. Sequences used for alignment and in the phylogenetic tree are: STM4563_SALTY: *S. typhimurium* NP_463419.1, YjjU_ECOLI: *E. coli* BAE78366.1, RssA_ECOLI: *E. coli* NP_415750.2, STM1754_SALTY: *S. typhimurium* YchK NC_003197.2, PA1640_PSEAI: *Pseudomonas aeruginosa* SG17M Homolog_EWH27047.1, CapV_ECOR31: *E. coli* ECOR31 OII97420.1, CapV_VIBCH: *V. cholerae* NC_002505.1, PLP2_SOLTU: *Solanum tuberosum*, PA3241_PSEAI: *P. aeruginosa* SG17M Homolog_EWH29020.1, PatA_LEGPH: *Legionella* lpg2317 NC_002942.5, RssA_HALMT: *Haloferax mediterranei* WP_004060664.1, Q68WK1_RICTY: *Rickettsia typhi* RT0522 WP_011190972.1, VpdB_LEGPH: *Legionella pneumophila* QGK66366.1, ExoU_PSAER: *P. aeruginosa* WP_003134060.1, VpdC_LEGPH: *L. pneumophila* QGK66518.1, VpdA_LEGPH: *L. pneumophila* CCD09784.1, VipD_LEGPH: *L. pneumophila* WP_010948518.1, CapE_ECOR31: *E. coli* ECOR31 OII97423.1, COTR_BACSU: *Bacillus subtilis* WP_003243674.1.

### Host cell adhesion assay-normalized values

Bacterial adhesion was assessed with the human uroepithelial cell line T24 cultured in McCoy’s 5a medium supplemented with 10% fetal bovine serum. *E. coli* No. 12 harboring pCapV, pCapV_Q329R_, or vector control pBAD28 were plated on Luria-Bertani agar plates and incubated at 37 °C overnight. The following day, one colony was inoculated in LB with 100 µg/ml of ampicillin and incubated overnight at 37 °C with 200 RPM. For induction of filaments, the cell suspension was diluted 10 times and subcultured in TB broth supplemented with 0.1% l-arabinose and 100 µg/ml of ampicillin at 200 RPM for 2 h. Microscopic observation confirmed filament formation. Infection of T24 host cells was carried out in 24-well cell culture plates (Costar) with 2 × 10^6^ CFU/ml (MOI 10) of *E. coli* No. 12 harboring pCapV, pCapV_Q329R_, or the vector control pBAD28 to be added to the cell culture in serum- and antibiotic-free medium and incubated at 37 °C. After 30 min, cells were washed three times with 37 °C warm PBS to remove non-adherent bacteria. To collect cell-associated bacteria, ice-cold PBS with 1% Triton X-100 was added. Lysates were plated on blood agar plates after serial dilution in PBS and bacterial numbers were counted after overnight incubation at 37 °C. The relative adhesion of *E. coli* No. 12 with pCapV or pCapV_Q329R_ was calculated in relation to vector control.

### Total RNA isolation and quantitative real-time PCR (qPCR) of host cells

The human uroepithelial cell line T24 was infected by co-incubation with isogenic strains of *E. coli* for 2 h^99^. Total RNA was extracted using the RNeasy Mini kit (Qiagen) according to the manufacturer’s protocol. The concentration and purity of RNA were determined with nanodrop, and 300 ng of RNA was transcribed to cDNA using the High-Capacity cDNA Reverse Transcription Kit (Applied Biosystems). Real-time PCR for human *IL1B* (forward 5′-CAC GAT GCA CCT GTA CGA TCA-3′, reverse 5′-GTT GCT CCA TAT CCT GTC CCT-3′), *CXCL8* (forward 5′-AAG AGA GCT CTG TCT GGA CC-3′, reverse 5′-GAT ATT CTC TTG GCC CTT GG-3′) were analyzed in comparison to housekeeping gene *ACTB* (forward 5′- AAG AGA GGC ATC CTC ACC CT-3′, reverse 5′-TAC ATC GCT GGG GTG TTG-3′) using SYBR green gene expression assay (Applied Biosystems) in a Rotor-Gene PCR cycler (Corbett Life Science). Relative expressions of target genes was presented as 2^−∆CT^ and fold change as 2^−∆∆CT^ compared to vector control infected cells.

### DRaCALA assay

Bacterial cells were prepared for the DRaCALA assay basically as described^100^. Cells were cultured overnight and subcultured to a starting OD_600_ of 0.02 induced with 0.1% arabinose. At OD600 = 2, cells were collected resuspended in 10 mM Tris, pH 8.0, 100 mM NaCl, and 5 mM MgCl_2._ Cells were lysed three times by freeze-thaw and the lysate was tested for binding ^32^P labelled cAMP-GMP starting with a fourfold dilution. ^32^P-cAMP-GMP had been prepared by mixing 10 µCi of ^32^P-α-GTP (8 nmol) and 250 µM ATP followed by addition of 10 µM DncV in reaction buffer (50 mM 3-(cyclohexylamino)-2-hydroxy-1-propanesulfonic acid (CAPSO) pH 9.4, 175 mM KCl, 25 mM, 175 mM Mg(OAc)_2_, and 5 mM DTT) and incubated for 2 h at 37 °C.

## Supplementary information

Supplementary Information

Movie 1a

Movie 1b

Movie 1c

Movie 1d

Movie 1e

Movie 1f

Movie 2a

Movie 2b

Movie 3a

Movie 3b

Movie 3c

## Data Availability

The data underlying this article are available in the article and in its online Supplementary Information file. Material such as bacterial strains and plasmids related to this paper may be requested from the corresponding author (Ute.Romling@ki.se) or from Addgene under the ID:80770. Lipidomics data have been made available at the Center for Open Science webpage under https://osf.io/aj58e/.
