# Supplementary material for: Patatin-like phospholipase CapV in Escherichia coli - morphological and physiological effects of one amino acid substitution
Source: NPJ Biofilms Microbiomes. 2022 May 11;8:39. doi: 10.1038/s41522-022-00294-z (PMC9095652; doi:10.1038/s41522-022-00294-z)

Supplementary Information

Patatin-like phospholipase CapV in *Escherichia coli* - morphological and physiological effects of one amino acid substitution

Short title: CapV variant expression in Escherichia coli

Fengyang Li[1,*], Lianying Cao[1], Heike Bähre[2], Soo-Kyoung Kim[3], Kirsten Schroeder[4], Kristina Jonas[4], Kira Koonce[4], Solomon A. Mekonnen[3], Soumitra Mohanty[5], Fengwu Bai[6], Annelie Brauner[5], Vincent T. Lee[3], Manfred Rohde[7], Ute Römling[1,*]

Affiliations

[1]Department of Microbiology, Tumor and Cell Biology, Karolinska Institutet, Stockholm, Sweden

[2]Research Core Unit Metabolomics, Hannover Medical School, Hannover, Germany

[3]Department of Cell Biology and Molecular Genetics, University of Maryland, College Park, Maryland 20742, United States

[4]Science for Life Laboratory, Department of Molecular Biosciences, The Wenner-Gren Institute, Stockholm University, Stockholm, Sweden

[5]Department of Microbiology, Tumor and Cell Biology, Division of Clinical Microbiology, Karolinska Institutet and Karolinska University Hospital, 17176 Stockholm, Sweden

[6] School of Life Sciences and Biotechnology, Shanghai Jiao Tong University, Shanghai, 200240 China

[7]Central Facility for Microscopy, Helmholtz Center for Infection Research, Braunschweig, Germany

*Current address: College of Veterinary Medicine, Jilin University, Changchun, China

*Address correspondence to Ute Römling (ute.romling@ki.se) or Fengyang Li (fylee1987@outlook.com)

Supplementary Information – Content **page**

Supplementary Results 4

Supplementary Figures

Fig. S1. Overexpression of the variant four gene operon *capV-dncV-vc0180-vc0181* cloned in pBAD28 (p*78901*) downregulates swimming motility and FliC production of *E. coli* MG1655. 5

Fig. S2. $CapV_{Q329R}$ of *E. coli* ECOR31, but not other variant gene products encoded by p*78901v* downregulate apparent swimming motility of MG1655. 6

Fig. S3. $CapV_{Q329}$ production is critical for induction of apparent inhibition of swimming motility in *E. coli* MG1655. 8

Fig. S4. Phylogenetic position and bioinformatic analysis of CapV among homologs. 9

Fig. S5. Lipidomic analysis of *E. coli* MG1655 overexpressing CapV and $CapV_{Q329R}$ revealed distinct lipidome profiles. 12

Fig. S6. $CapV_{Q329R}$-induced cell filamentation is independent of the SOS gene *sulA* and does hardly affect FtsZ and FtsA production. 13

Fig. S7: Overproduction of CapV and $CapV_{Q329R}$ in *E. coli* MG1655 restricts cell viability as assessed by the LIVE/DEAD™ BacLight™ Viability Kit. 16

Fig. S8. Effects of various B-vitamins on $CapV_{Q329R}$-induced cell filamentation of *E. coli* MG1655. 18

Fig. S9. Overexpression of $CapV_{Q329R}$ in *E. coli* MG1655 restricts apparent swimming motility, induces cell filamentation and alters rdar biofilm formation of a panel of commensal and UPEC *E. coli* strains and *S. typhimurium* UMR1. 19

Fig. S10. Phage sensitivity, antimicrobial susceptibility and host cell interaction phenotypes mediated by $pCapV_{Q329R}$ in *E. coli* MG1655 and UPEC *E. coli* No. 12. 21

Supplementary Tables

Table S1. Bacterial strains and plasmids used in this study 22

Table S2. Primer used in this study. 24

Table S3 Plasmids used to assess effect of mutations in p*78901v*. 26

Table S4 Primers for qRT-PCR of bacterial genes. 27

Information about Supplementary material not included in this file 28
Supplementary movies
Movie S1. *E. coli* MG1655 cells examined by light microscopy 4 and 6 h after induction of production of wild type CapV and $\text{CapV}_{\text{Q329R}}$ by 0.1% L-arabinose in TB medium at 37 °C.

Movie S2. *E. coli* MG1655 cells examined under a light microscope 22 h after induction of $\text{CapV}_{\text{Q329R}}$ production by 0.2% L-arabinose in TB medium at 37 °C.

Movie S3. Time-lapse analysis of *E. coli* MG1655 *mCherry-minC* expressing cells with pBAD28 vector control (a), pCapV (b) and $\text{pCapV}_{\text{Q329R}}$ (c).

References 29

Additional data – uncropped gels and blots 30

Supplementary results

The dinucleotide cyclase DncV synthesizes cAMP-GMP to inhibit rdar biofilm formation and motility in the animal commensal strain *E. coli* ECOR31 (1). *DncV* is flanked by *V. cholerae* 7th pandemic island-1 (VSP-1) homologs; *capV* upstream and *vc0180* and *vc0181* downstream (1-3) (Supplementary Fig. 1a). These genes constitute a 4.6 kbp four-gene cluster which contributes to differential functionality of *dncV* in *E. coli* ECOR31 (1). To understand physiological functions of the putative four-gene operon (designed *78901*), we had previously cloned the four genes in pBAD28 under the regulation of the pBAD promoter (1). In this work, we tested their combined effect on swimming motility in semi-solid agar for the heterologous host *E. coli* K-12 derivative MG1655. Overexpression of the cloned four-gene operon (designated *78901v*, see below) from the pBAD promoter in the pBAD28 plasmid significantly inhibited swimming motility at 37 °C and 28 °C, as did five other independent clones (Supplementary Fig. 1b and c and data not shown). Since the plate assay monitors both chemotaxis and motility, we assessed production of cell associated flagellin FliC upon overexpression of *78901v*. While *E. coli* MG1655 pronouncedly produced flagellin, which stems from flagella, polymerized monomeric flagellin, overexpression of *78901v* inhibited flagellin production (Supplementary Fig. 1d).

The first ORF of *capV-dncV-vc0180-vc0181* is *capV* (Supplementary Fig. 1a). CapV possesses a N-terminal patatin-like phospholipase A2 (PNPLA) domain, the enzymatic activity of which is stimulated by cAMP-GMP in *V. cholerae* (4). *VC0180* encodes an eukaryotic-like ubiquitin ligase with ThiF (E1)/ E2 domains and *VC0181*, an isopeptidase with a JAB domain, respectively (3, 4) (Supplementary Fig. 2a). To clarify which gene in the putative four-gene operon contributes to inhibition of *E. coli* MG1655 swimming motility, we constructed amino acid substitutions in the catalytic motif of each of the gene products. Expression of the four genes from p*78901v* (Table S1) with an amino acid substitution in the D-G-[A/G] motif of CapV (leading to *78901v*$_{D197A}$) did not suppress apparent swimming motility nor cell-associated and secreted flagellin production, while substitutions in residues involved in the catalysis of other proteins maintained the suppressive effect (Supplementary Fig. 1d and 2b-e).

Surprisingly, both overexpression of wild type *capV* and *dncV*-vc0180-vc0181* (p79801v)*

had no effect on apparent swimming motility of *E. coli* MG1655 (Supplementary Fig. 3a). Subsequently, sequence analysis showed the presence of six mutations in the cloned insert of p*78901v*, whereby the nucleotide alternation in the *capV* open reading frame led to the substitution of glutamine 329 by arginine (Q329R) (Supplementary Fig. 3a and c). Three additional nucleotide alterations provide non-synonymous mutations (besides Q329R, leading to K102R and L275P in DncV and, putatively, R6K in VC0181). To investigate the molecular basis of apparent swimming repression of p*78901v*, we constructed a plasmid that expressed the CapV$_{Q329R}$ variant, which suppressed apparent swimming motility. Moreover, only reversion of the Q329R mutation in construct p*78901v* expressing the four genes resulting to p*78901v*$_{R329Q}$ relieved swimming repression, while nucleotide substitutions reverting the non-synonymous mutations in *dncV* and *vc0181* failed to relieve swimming repression (Supplementary Fig. 3b, d, e).

Supplementary Figures

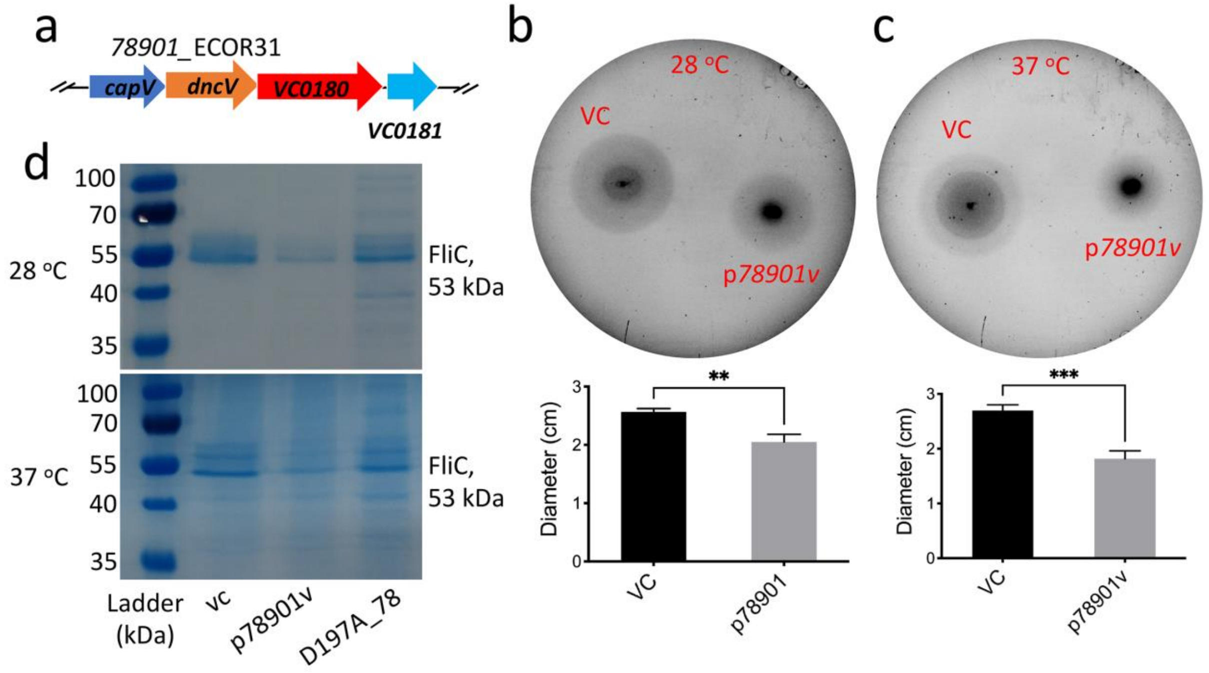

Supplementary Figure 1. Overexpression of the variant four gene operon *capV- dncV-vc0180-vc0181* cloned in pBAD28 (p*78901v*) (a) downregulates swimming motility (b and C) and FliC production (d) of *E. coli* MG1655. Three µl of OD_{600} = 5 cells were inoculated into soft agar plates containing 1% tryptone, 0.5% NaCl and 0.25% agar and the swimming diameter was measured after 8 h at 28 °C and 6 h at 37 °C. Bars represent mean values with error bars to represent standard deviation (SD) from three independent replicates. Differences between mean values were assessed by two-tailed Student's *t*-test (**$p < 0.01$, and ***$p < 0.001$ compared to MG1655 vector control). VC = pBAD28, p*78901v* = $capV_{Q329R}$-*dncV*-vc0180-vc0181** cloned in pBAD28, p*78901v*$_{D197A}$ = $capV_{Q329R/D197A}$-*dncV*-vc0180-vc0181** cloned in pBAD28.

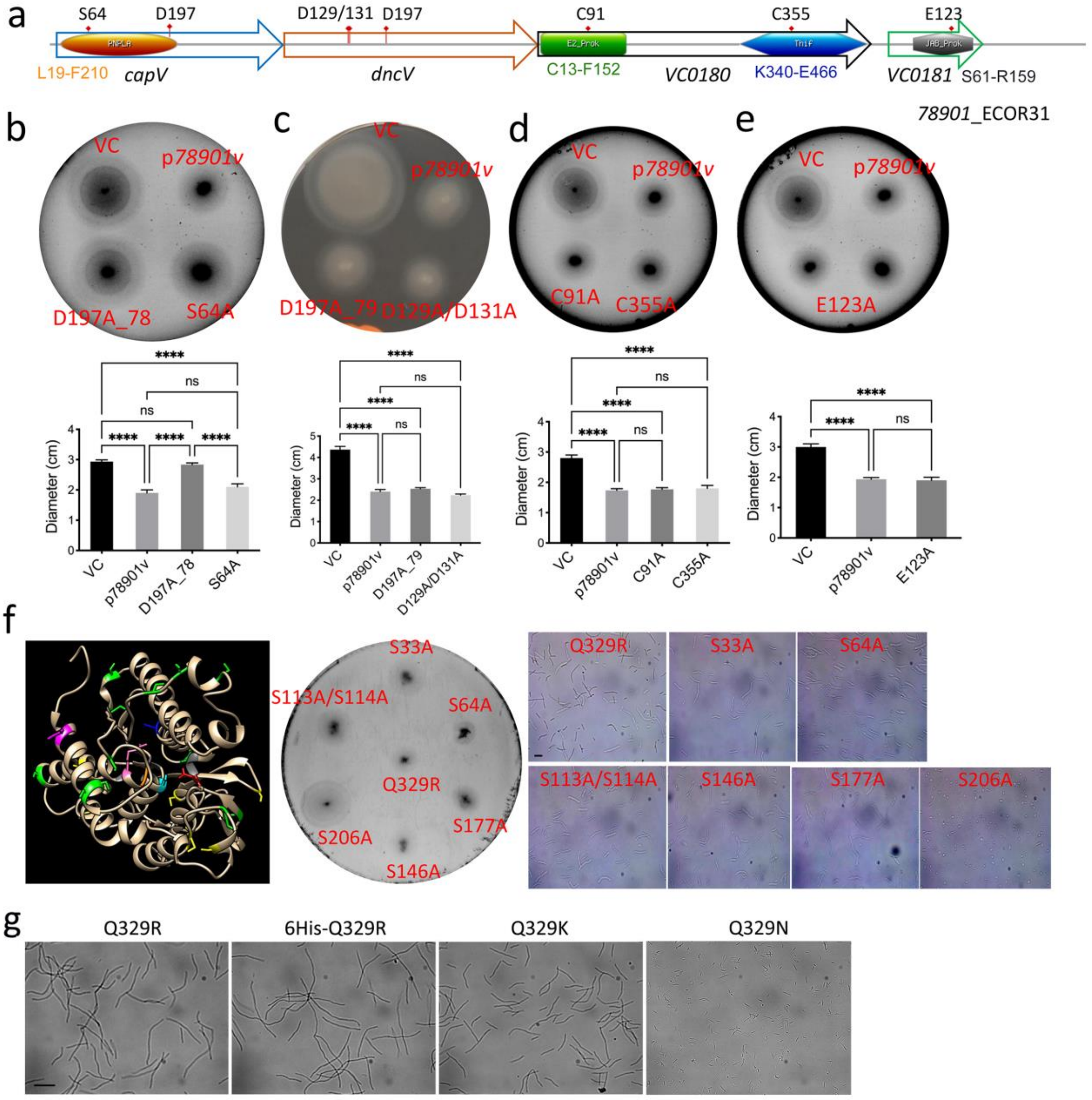

Supplementary Figure 2. CapV$_{Q329R}$ of p*78901v*, but not other variant gene products encoded by p*78901v* downregulate apparent swimming motility of MG1655 a Schematic illustration of *E. coli* ECOR31 four gene operon with domain profiles of gene products. The putative catalytic residues are indicated by filled red triangles. Domain prediction was performed with the InterPro server, and the result was processed with ExPASy_Prosite_-MyDomains. **b-e** Effects of aa substitutions in the catalytic motifs of p*78901v* encoded gene products on apparent swimming motility of *E. coli* MG1655 Three µl of OD_{600} = 5 cells were inoculated into soft agar plates containing 1% tryptone, 0.5% NaCl and 0.25% agar and the swimming diameter was measured after 6 h at 37 °C. VC = pBAD28, p*78901v* = *capV$_{Q329R}$-dncV*-vc0180-vc0181** cloned in pBAD28. pCapV = CapV cloned in pBAD28; S64A = *capV$_{Q329R/S64A}$-dncV*-vc0180-vc0181** cloned in pBAD28. D197A_78 = *capV$_{Q329R/D197A}$-dncV*-vc0180-vc0181** cloned in pBAD28. D197A_79= *capV$_{Q329R}$-dncV$_{K102R/D197A/L275P}$-vc0180-vc0181** cloned in pBAD28. D129A/131A= *capV$_{Q329R}$-dncV$_{K102R/D129A/D131A/L275P}$-vc0180-vc0181** cloned in pBAD28. C91A = *capV$_{Q329R}$-dncV*-vc0180$_{C91A}$-vc0181** cloned in pBAD28. C355A = *capV$_{Q329R}$-dncV*-vc0180$_{C355A}$-vc0181**

cloned in pBAD28. E123A = *capV*$_{Q329R}$*-dncV*-vc0180-vc0181*$_{E123A}$ cloned in pBAD28. Bars represent mean values with error bars to represent SD from three independent replicates. Differences between mean values were assessed by two-tailed Student's *t*-test (ns, not significant and ***p < 0.001 compared to *E. coli* MG1655 vector control). **f** Effects of aa substitutions of CapV$_{Q329R}$ close to the catalytic site on apparent swimming motility and filamentation of *E. coli* MG1655. Structural model of CapV$_{Q329R}$ with catalytic and substituted aa indicated (catalytic Arg27, light pink; catalytic Ser64 light blue; catalytic Asp197: red; Gly24 and Gly25, brown; Arg329, pink; Ser not mutated, green; Ser mutated no effect; yellow; Ser 206, dark blue. Amino acids of the characteristic motifs not mutated: dark grey. Q329R = CapV$_{Q329R}$ cloned in pBAD28. S64A = CapV$_{Q329R/S64A}$ cloned in pBAD28. S113A/S114A = CapV$_{Q329R/\ S113A/S114A}$ cloned in pBAD28. S146A = CapV$_{Q329R/S146A}$ cloned in pBAD28. S177A = CapV$_{Q329R/S177A}$ cloned in pBAD28. S206A = CapV$_{Q329R/S206A}$ cloned in pBAD28. **g** Effects of the 6xHis-tag and aa substitutions of CapV$_{Q329R}$, CapV$_{Q329K}$ and CapV$_{Q329N}$ on filamentation of *E. coli* MG1655. 6His-Q329R = CapV$_{Q329R}$-6xHis cloned in pBAD28; R329K = CapV$_{R329K}$ cloned in pBAD28; R329N = CapV$_{R329N}$ cloned in pBAD28.

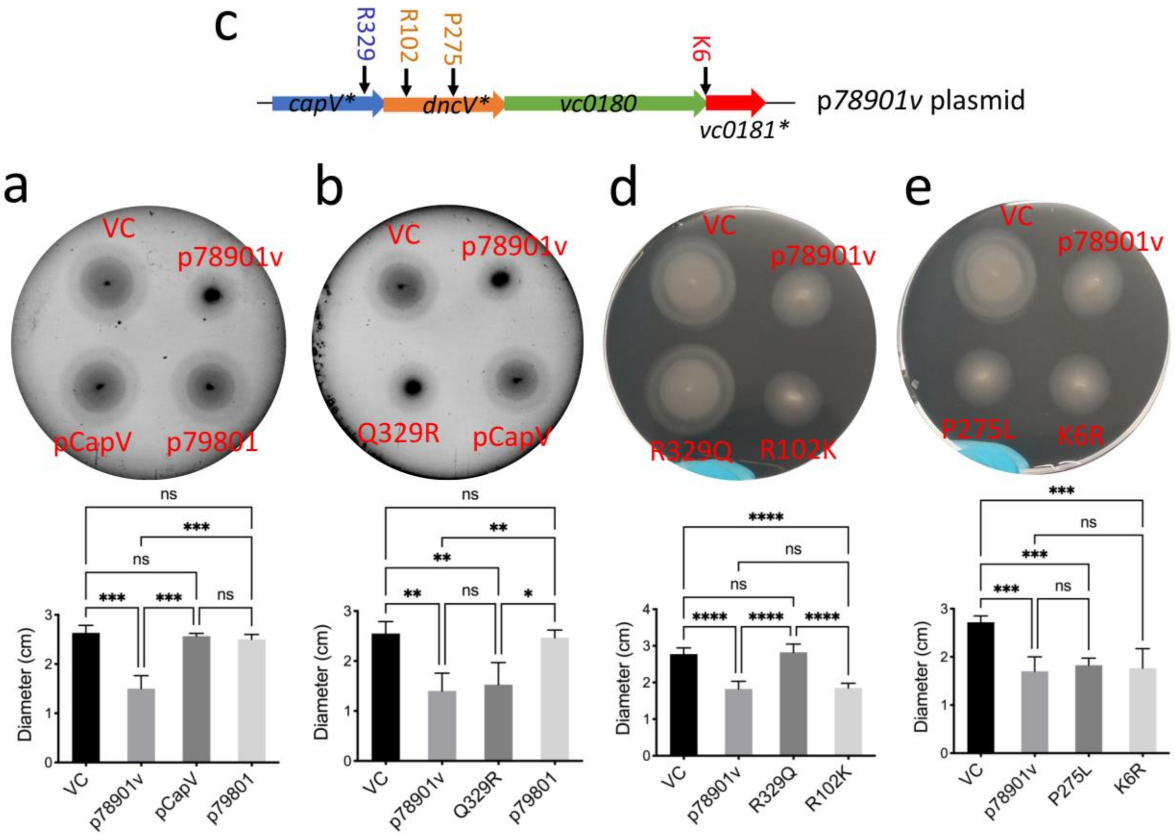

Supplementary Figure 3. CapV$_{Q329}$ production is critical for induction of apparent inhibition of swimming motility by p*78901v* or expressed as a stand-alone gene product in *E. coli* MG1655. **a** and **b**, Effects of the aa substitution in CapV upon dissection of the p*78901v* construct on apparent swimming motility of MG1655. p*78901v* represents *capV$_{Q329R}$-dncV*-vc0180-vc0181**; p*79801v* represents *dncV*-vc0180-vc0181** cloned in pBAD28. pCapV = CapV cloned in pBAD28. Q329R = CapV$_{Q329R}$ cloned in pBAD28. **c** Schematic illustration of the aa changes in the p*78901v* gene products compared to *78901* chromosomal sequence. **d** and **e** Effects of the aa substitution in DncV* and VC0181* on functionality of the p*78901v* construct for apparent swimming motility of *E. coli* MG1655. R329Q = *capV-dncV*-vc0180-vc0181** cloned in pBAD28. R102K = *capV$_{Q329R}$-dncV*$_{R102K}$-vc0180-vc0181** cloned in pBAD28; P275L = *capV$_{Q329R}$-dncV*$_{P275L}$-vc0180-vc0181** cloned in pBAD28; K6R = *capV$_{Q329R}$-dncV*-vc0180-vc0181* cloned in pBAD28.

Three µl of OD_{600} = 5 cell suspension was inoculated into soft agar plates containing 1% tryptone, 0.5% NaCl and 0.25% agar and the swimming diameter was measured after 6 h at 37 °C.

Bars represent mean values with error bars to represent SD from three independent replicates. Differences between mean values were assessed by two-tailed Student's *t*-test (ns, not significant; *$p < 0.05$, **$p < 0.01$, and ***$p < 0.001$ compared to *E. coli* MG1655 vector control). VC = pBAD28.

a

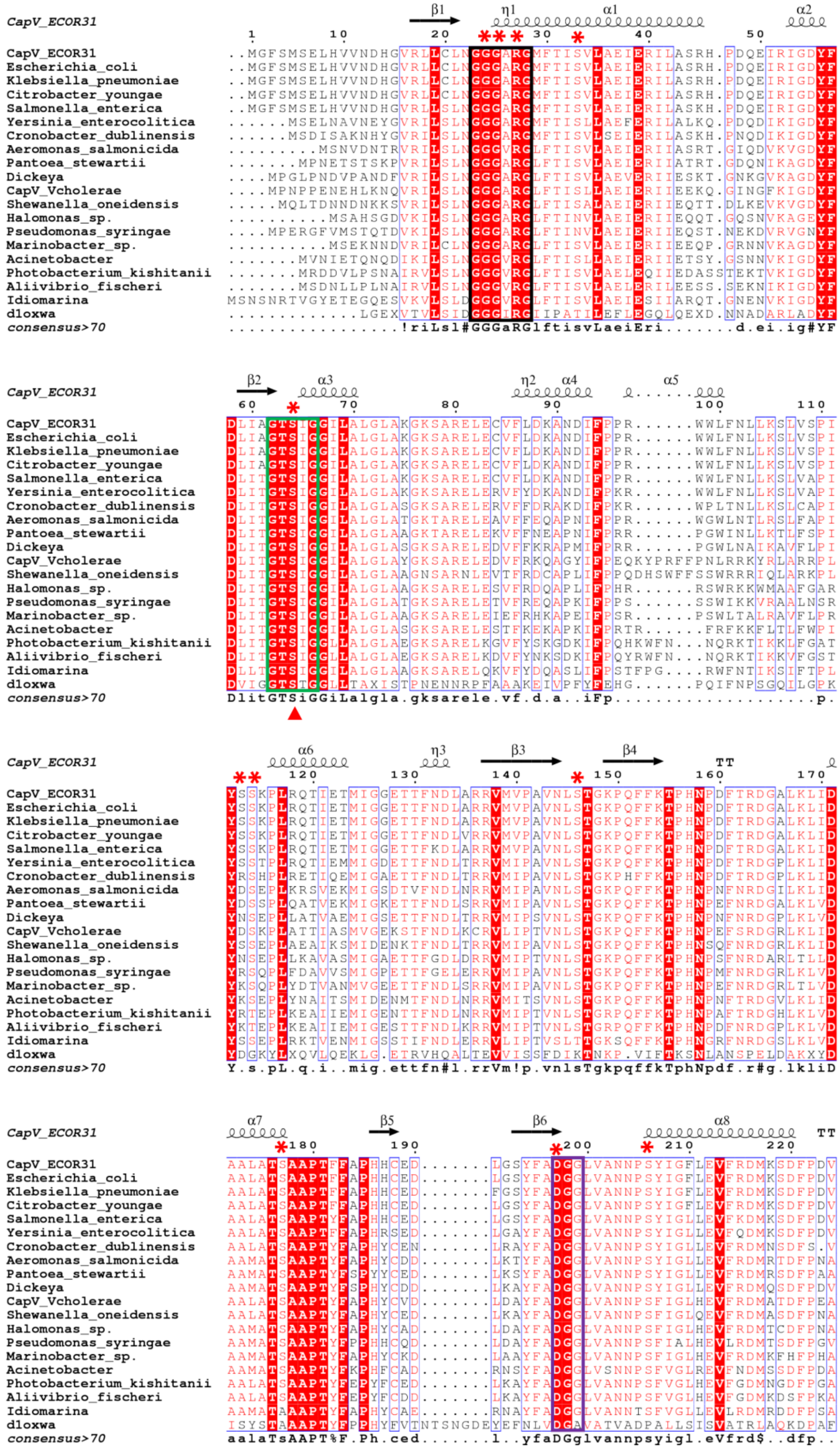

CapV_ECOR31
β1 η1 α1 α2
β2 α3 η2 α4 α5
α6 η3 β3 β4 TT
α7 β5 β6 α8 TT
CapV_ECOR31
Escherichia_coli
Klebsiella_pneumoniae
Citrobacter_youngae
Salmonella_enterica
Yersinia_enterocolitica
Cronobacter_dublinensis
Aeromonas_salmonicida
Pantoea_stewartii
Dickeya
CapV_Vcholerae
Shewanella_oneidensis
Halomonas_sp.
Pseudomonas_syringae
Marinobacter_sp.
Acinetobacter
Photobacterium_kishitanii
Aliivibrio_fischeri
Idiomarina
d1oxwa
consensus>70

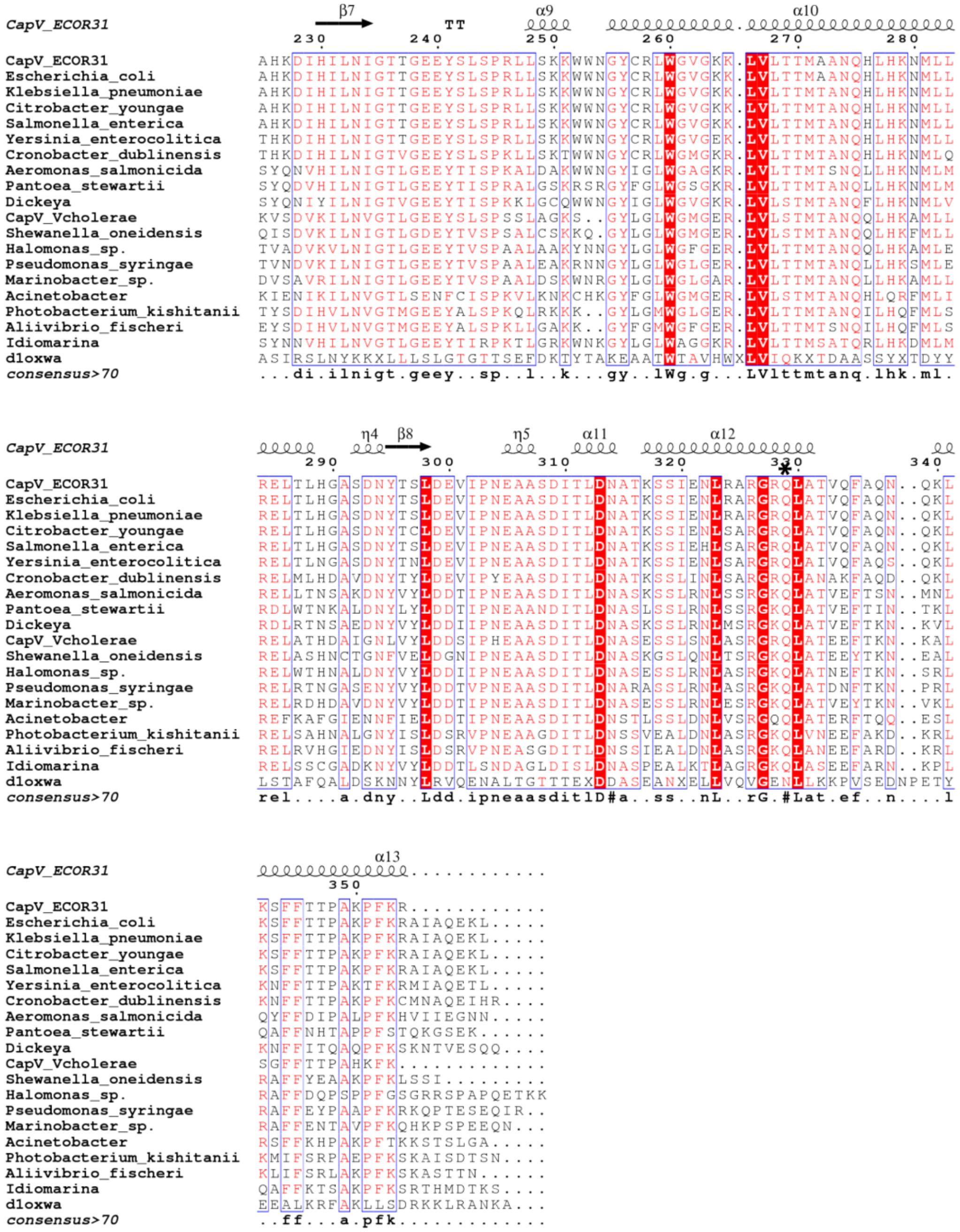
CapV_ECOR31 β7 TT α9 α10
230 240 250 260 270 280
CapV_ECOR31 AHKDIHILNIGTTGEEYSLSPRLLSKKWWNGYCRLWGVGKK.LVLTTMAANQHLHKNMLL
Escherichia_coli AHKDIHILNIGTTGEEYSLSPRLLSKKWWNGYCRLWGVGKK.LVLTTMAANQHLHKNMLL
Klebsiella_pneumoniae AHKDIHILNIGTTGEEYSLSPRLLSKKWWNGYCRLWGVGKK.LVLTTMTANQHLHKNMLL
Citrobacter_youngae AHKDIHILNIGTTGEEYSLSPRLLSKKWWNGYCRLWGVGKK.LVLTTMTANQHLHKNMLL
Salmonella_enterica AHKDIHILNIGTTGEEYSLSPRLLSKKWWNGYCRLWGVGKK.LVLTTMTANQHLHKNMLL
Yersinia_enterocolitica THKDIHILNIGTTGEEYSLSPRLLSKKWWNGYCRLWGVGEK.LVLTTMTANQHLHKNMLL
Cronobacter_dublinensis THKDIHILNIGTVGEEYSLSPKLLSKTWWNGYCRLWGMGKR.LVLTTMTANQHLHKNMLQ
Aeromonas_salmonicida SYQNVHILNIGTLGEEYTISPKALDAKWWNGYIGLWGAGKR.LVLTTMTSNQLLHKNMLM
Pantoea_stewartii SYQDVHILNIGTLGEEYTISPRALGSKRSRGYFGLWGSGKR.LVLTTMTANQLLHKNMLM
Dickeya SYQNIYILNIGTVGEEYTISPKKLGCQWWNGYIGLWGVGKR.LVLSTMTANQFLHKNMLV
CapV_Vcholerae KVSDVKILNVGTLGEEYSLSPSSLAGKS..GYLGLWGMGER.LVLSTMTANQQLHKAMLL
Shewanella_oneidensis QISDVKILNIGTLGDEYTVSPSALCSKKQ.GYLGLWGMGER.LVLSTMTANQQLHKFMLL
Halomonas_sp. TVADVKVLNIGTLGEEYTVSPAALAAKYNNGYLGLWGFGER.LVLTTMTANQQLHKAMLE
Pseudomonas_syringae TVNDVKILNIGTLGEEYTVSPAALEAKRNNGYLGLWGLGER.LVLTTMTANQLLHKSMLE
Marinobacter_sp. DVSAVRILNIGTLGEEYTVSPAALDSKWNRGYLGLWGLGAR.LVLTTMTANQLLHKAMLL
Acinetobacter KIENIKILNVGTLSENFCISPKVLKNKCHKGYFGLWGMGER.LVLSTMTANQHLQRFMLI
Photobacterium_kishitanii TYSDIHVLNVGTMGEEYALSPKQLRKKK..GYLGMWGLGER.LVLTTMTANQILHQFMLS
Aliivibrio_fischeri EYSDIHVLNVGTMGEEYALSPKLLGAKK..GYFGMWGFGER.LVLTTMTSNQILHQFMLS
Idiomarina SYNNVHILNVGTLGEEYTIRPKTLGRKWNKGYLGLWAGGKR.LVLTTMSATQRLHKDMLM
d1oxwa ASIRSLNYKKXLLSLGTGTTSEFDKTYTAKEAATWTAVHWXLVIQKXTDAASSYXTDYY
consensus>70 ...di.ilnigt.geey..sp..l..k...gy..lWg.g...LVlttmtanq.lhk.ml.

CapV_ECOR31 η4 β8 η5 α11 α12
290 300 310 320 330 340
CapV_ECOR31 RELTLHGASDNYTSLDEVIPNEAASDITLDNATKSSIENLRARGRQLATVQFAQN..QKL
Escherichia_coli RELTLHGASDNYTSLDEVIPNEAASDITLDNATKSSIENLRARGRQLATVQFAQN..QKL
Klebsiella_pneumoniae RELTLHGASDNYTSLDEVIPNEAASDITLDNATKSSIENLRARGRQLATVQFAQN..QKL
Citrobacter_youngae RELTLHGASDNYTCLDEVIPNEAASDITLDNATKSSIENLSARGRQLATVQFAQN..QKL
Salmonella_enterica RELTLHGASDNYTSLDEVIPNEAASDITLDNATKSSIEHLSARGRQLATVQFAQN..QKL
Yersinia_enterocolitica RELTLNGASDNYTNLDEVIPNEAASDITLDNATKSSIENLSARGRQLAIVQFAQS..QKL
Cronobacter_dublinensis RELMLHDAVDNYTYLDEVIPYEAASDITLDNATKSSLINLSARGRQLANAKFAQD..QKL
Aeromonas_salmonicida RELLTNSAKDNYVYLDDTIPNEAASDITLDNASKSSLRNLSSRGKQLATVEFTSN..MNL
Pantoea_stewartii RDLWTNKALDNYLYLDDTIPNEAANDITLDNASLSSLRNLSSRGKQLATVEFTIN..TKL
Dickeya RDLRTNSAEDNYVYLDDIIPNEAASDITLDNASKSSLRNLMSRGKQLATVEFTKN..KVL
CapV_Vcholerae RELATHDAIGNLVYLDDSIPHEAASDITLDNASESSLSNLASRGRQLATEEFTKN..KAL
Shewanella_oneidensis RELASHNCTGNFVELDGNIPNEAASDITLDNASKGSLQNLTSRGKQLATEEYTKN..EAL
Halomonas_sp. RELWTHNALDNYVYLDDIIPNEAASDITLDNASESSLRNLASRGKQLATNEFTKN..SRL
Pseudomonas_syringae RELRTNGASENYVYLDDTVPNEAASDITLDNARASSLRNLASRGKQLATDNFTKN..PRL
Marinobacter_sp. RELRDHDAVDNYVYLDDTIPNEAASDITLDNASESSLRNLESRGKQLATVEYTKN..VKL
Acinetobacter REFKAFGIENNFIELDDTIPNEAASDITLDNSTLSSLDNLVSRGQQLATERFTQQ..ESL
Photobacterium_kishitanii RELSAHNALGNYISLDSRVPNEAAGDITLDNSSVEALDNLASRGKQLVNEEFAKD..KRL
Aliivibrio_fischeri RELRVHGIEDNYISLDSRVPNEASGDITLDNSSIEALDNLASRGKQLANEEFARD..KRL
Idiomarina RELSSCGADKNYVYLDDTLSNDAGLDISLDNASPEALKTLAGRGKQLASEEFARN..KPL
d1oxwa LSTAFQALDSKNNYLRVQENALTGTTTEXDDASEANXELLVQVGENLLKKPVSEDNPETY
consensus>70 rel....a.dny..Ldd.ipneaasditlD#a..ss..nL..rG.#Lat.ef..n....l

CapV_ECOR31 α13
350
CapV_ECOR31 KSFFTTPAKPFKR...........
Escherichia_coli KSFFTTPAKPFKRAIAQEKL.....
Klebsiella_pneumoniae KSFFTTPAKPFKRAIAQEKL.....
Citrobacter_youngae KSFFTTPAKPFKRAIAQEKL.....
Salmonella_enterica KSFFTTPAKPFKRAIAQEKL.....
Yersinia_enterocolitica KNFFTTPAKTFKRMIAQETL.....
Cronobacter_dublinensis KNFFTTPAKPFKCMNAQEIHR....
Aeromonas_salmonicida QYFFDIPALPFKHVIIEGNN.....
Pantoea_stewartii QAFFNHTAPPFSTQKGSEK......
Dickeya KNFFITQAQPFKSKNTVESQQ....
CapV_Vcholerae SGFFTTPAHKFK............
Shewanella_oneidensis RAFFYEAAKPFKLSSI........
Halomonas_sp. RAFFDQPSPPFGSGRRSPAPQETKK
Pseudomonas_syringae RAFFEYPAAPFKRKQPTESEQIR..
Marinobacter_sp. RAFFENTAVPFKQHKPSPEEQN...
Acinetobacter RSFFKHPAKPFTKKSTSLGA.....
Photobacterium_kishitanii KMIFSRPAEPFKSKAISDTSN....
Aliivibrio_fischeri KLIFSRLAKPFKSKASTTN......
Idiomarina QAFFKTSAKPFKSRTHMDTKS....
d1oxwa EEALKRFAKLLSDRKKLRANKA...
consensus>70 ..ff...a.pfk............

b

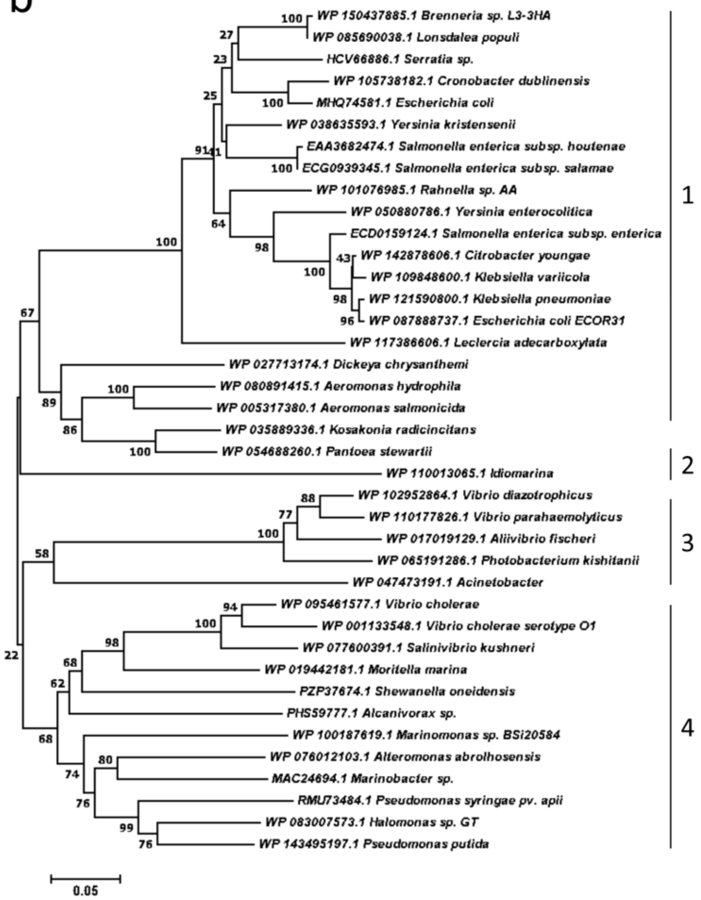

Supplementary Figure 4. Phylogenetic position and bioinformatic analysis of CapV among homologs. a Alignment of CapV from *E. coli* ECOR31, *V. cholerae biovar* El Tor and other species with >60% sequence identity. The putative secondary structures of CapV from homology modeling are shown above the alignments. The conserved motifs of the PNPLA domain, G-G-G-x-[K/R]-G, G-x-S-x-G, and D-G-[A/G], boxed in black, green, and purple, respectively. Completely conserved residues are shown in white on a red background. Conserved residues are boxed. The putative catalytic residues of CapV are indicated with filled red triangles. $CapV_{Q329}$ is marked with a black asterisk above the sequence. The residues in $CapV_{Q329R}$ mutated to alanine are marked with red asterisks above the sequence. The consensus sequence below the alignment indicates in uppercase residues with 100% conservation and in lowercase residues higher than >70% conservation. Alignment was performed using CLUSTALW using standard parameters (5), and the result processed with ESPript 3.0 (6). **b** Maximum likelihood phylogenetic reconstruction of CapV homologs. CapV from *E. coli* ECOR31 was used as the query in the NCBI Blast search and representative proteins (one representative per genus of equal homology) with >60% aa identity over the entire length of the protein sequence were retrieved.

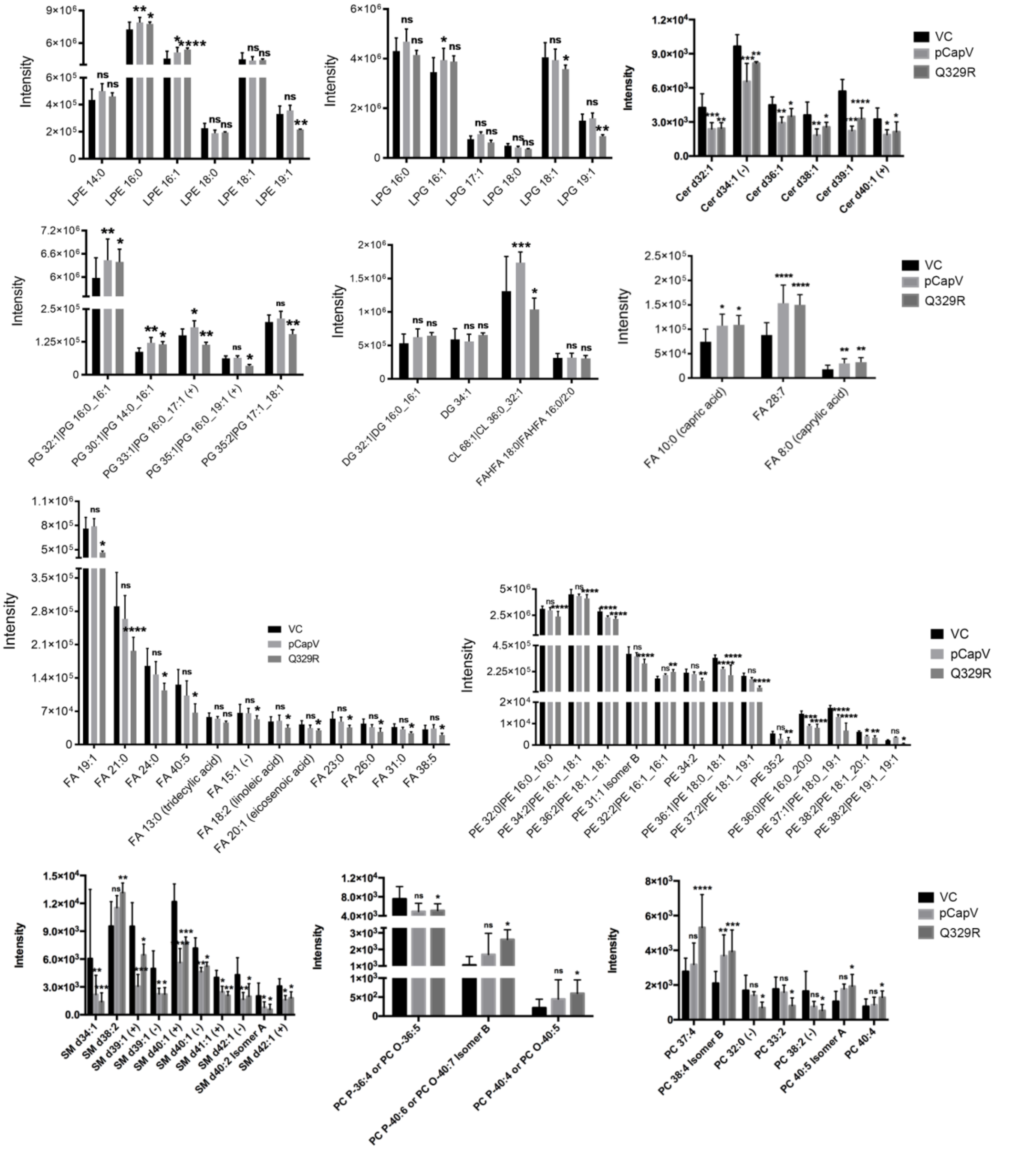

Supplementary Figure 5. Lipidomic analysis of *E. coli* MG1655 overexpressing CapV and $\text{CapV}_{\text{Q329R}}$ revealed distinct lipidome profiles. Relative abundance of Cer, ceramide; LPG, lysophosphatidylglycerol; SM, sphingomyelin; PC, phosphatidylcholine; LPE, lysophosphatidylethanolamine; DG, diacylglycerol; PG, phosphatidylglycerol; PE, phosphatidylethanolamine; and FA, fatty acids derivatives by untargeted CSH-QTOF MS analysis. Bars represent mean values from five independent replicates with error bars to represent SD. Differences between mean values were assessed by two-tailed Student's *t*-test (ns, not significant; *$p < 0.05$, **$p < 0.01$, and ***$p < 0.001$ compared to MG1655 VC).

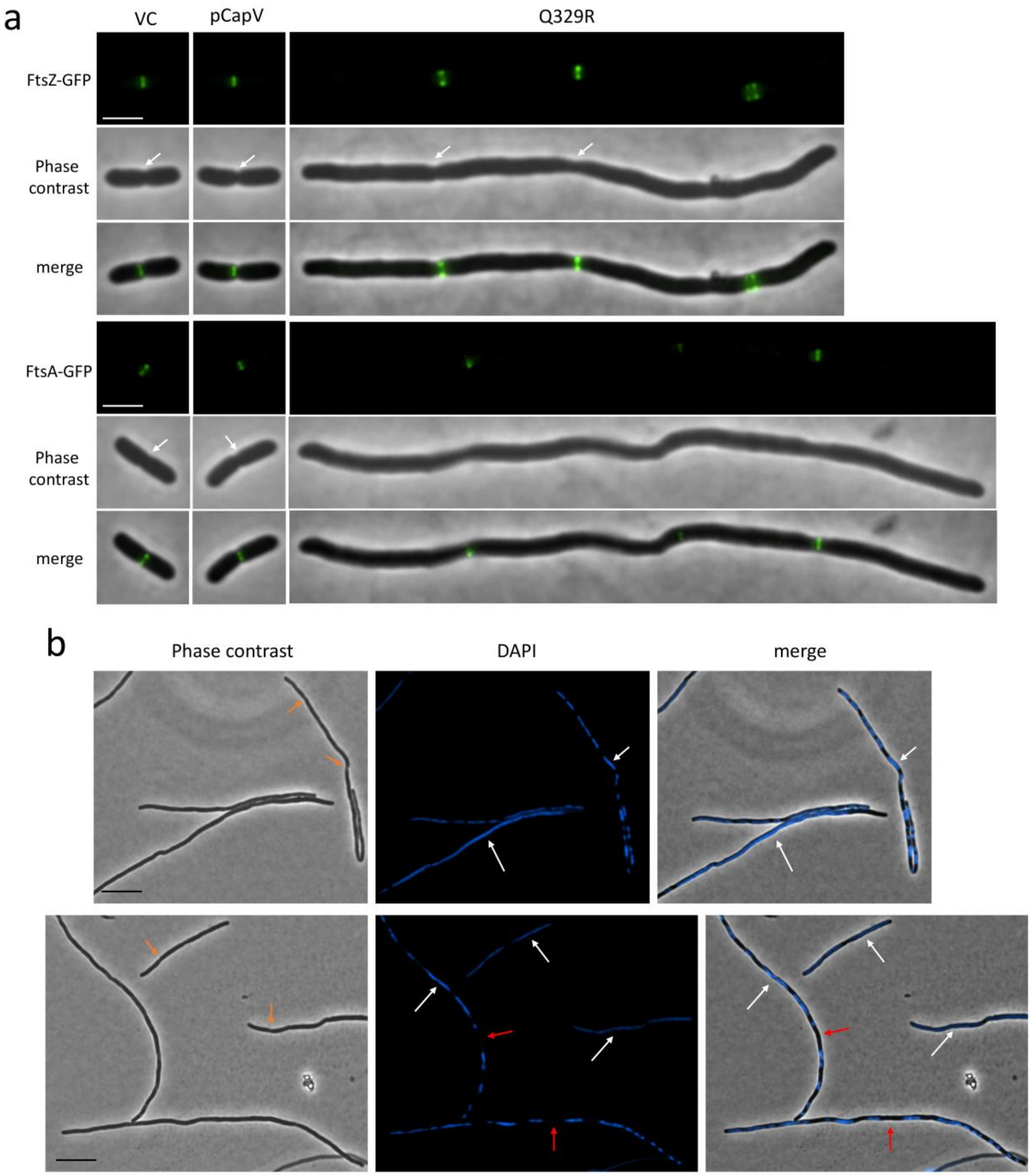
a
VC
pCapV
Q329R
FtsZ-GFP
Phase contrast
merge
FtsA-GFP
Phase contrast
merge
b
Phase contrast
DAPI
merge

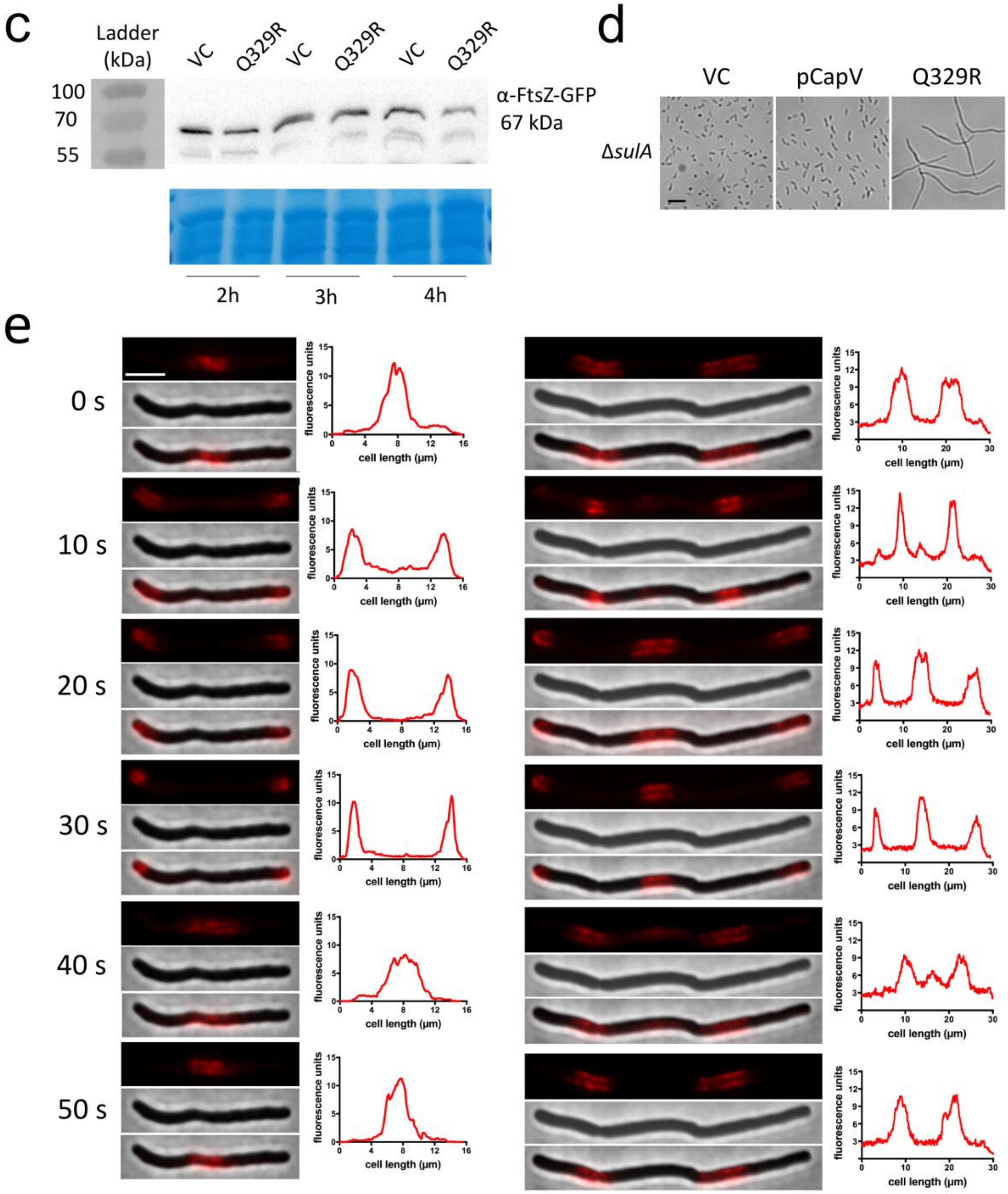

Supplementary Figure 6. CapV$_{Q329R}$-induced cell filamentation is independent of the SOS gene *sulA* and does hardly affect FtsZ and FtsA production. a Cells were grown for 4 h at 37 °C and placed on an agarose pad to be observed by fluorescence microscopy. Septa are indicated by white arrows. Bar, 3 µm. **b** Chromosomal segregation is impaired in filamenting cells. Cells were cultured in TB medium at 37 °C for 4 h, stained with DAPI and assessed under fluorescence microscopy immediately. Large fragments of unsegregated nucleoids are indicated by white arrows. Suspected septa are indicated by orange arrows. Large spaces between nucleoids are indicated by red arrows. Bar, 5 µm. **c** Cells expressing FtsZ-GFP protein were grown in TB medium at 37 °C, and samples were harvested after 2 h, 3 h, and 4 h of growth for Western blot analysis. **d** CapV$_{Q329R}$-induced cell filamentation is *sulA*-independent. Light microscopy pictures of cell filamentation in an *E. coli* MG1655 Δ*sulA* mutant after 4 h incubation at 37 °C imaged by light

microscopy. Bar, 5 µm. **e** Time-lapse analysis of mCherry-MinC expressing cells (PB318) upon $CapV_{Q329R}$ overexpression (see also Fig. 5c). A representative elongating cell is displayed. Graphs on the right of the fluorescence images display the line profiles of fluorescent signals emanating from the cell. Arbitrary fluorescent units are obtained, analyzed by the Fiji ImageJ 1.8.0 software and plotted on the y-axis; cell length in µm is plotted on the x-axis. Bar, 3 µm. VC = pBAD28; CapV = wild type CapV cloned in pBAD28; $CapV_{Q329R}$ = mutant $CapV_{Q329R}$ cloned in pBAD28.

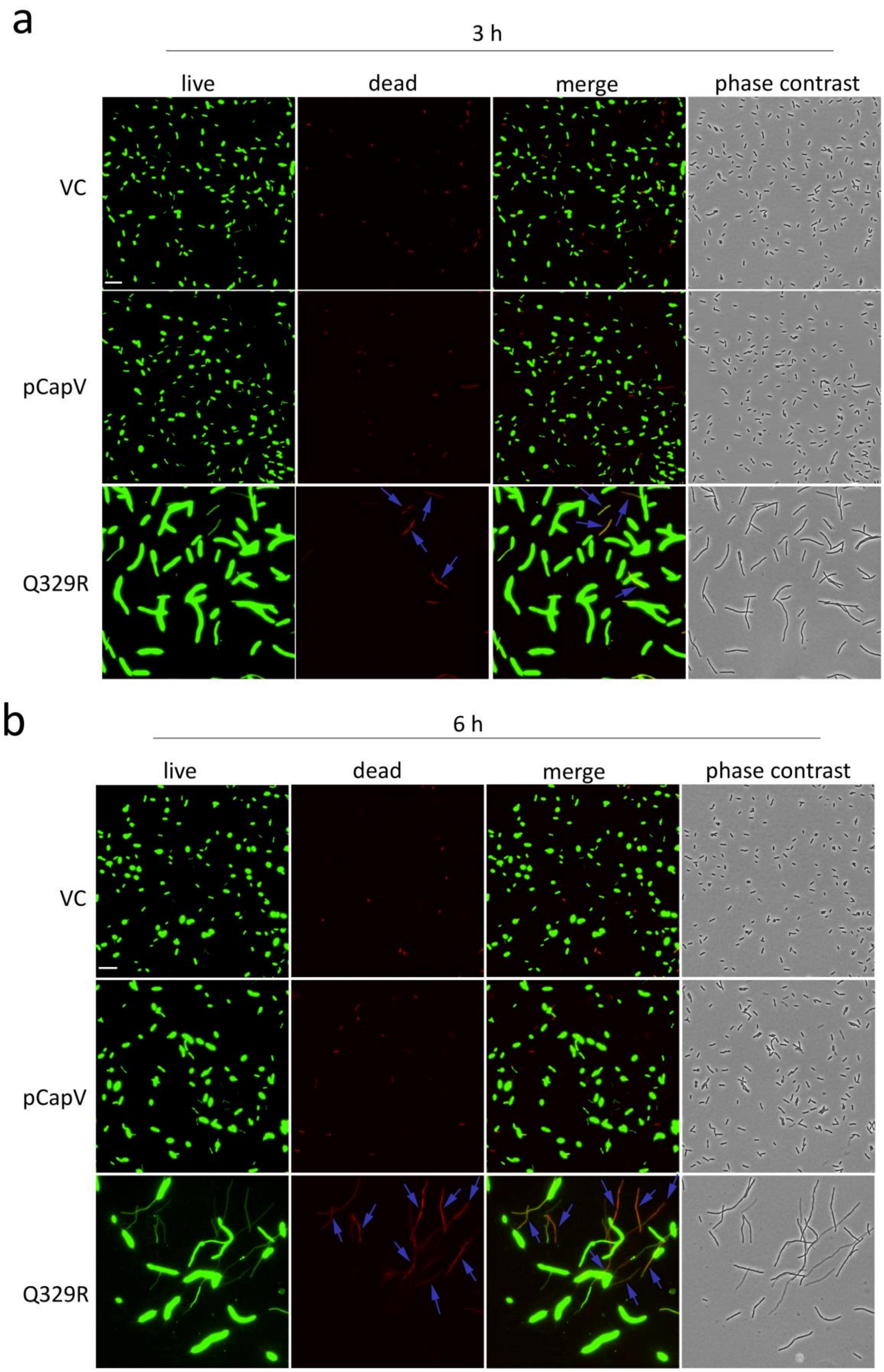
a
3 h
live
dead
merge
phase contrast
VC
pCapV
Q329R
b
6 h
live
dead
merge
phase contrast
VC
pCapV
Q329R

Supplementary Figure 7. Overproduction of CapV and CapV$_{Q329R}$ in *E. coli* MG1655 restricts cell viability as assessed by the LIVE/DEAD™ BacLight™ Viability Kit. Cells were incubated for 3 h and 6 h at 37 °C and stained with SYTO 9 and propidium iodide (PI). The cell viability was analyzed under a fluorescence microscope. Green, SYTO9 uptake/live cells. Red, PI uptake/dead cells. Blue arrows, uptake of SYTO9 and PI. Bar, 5 µm. VC = pBAD28; CapV = wild type *capV* cloned in pBAD28; CapV$_{Q329R}$ = mutant CapV$_{Q329R}$ cloned in pBAD28.

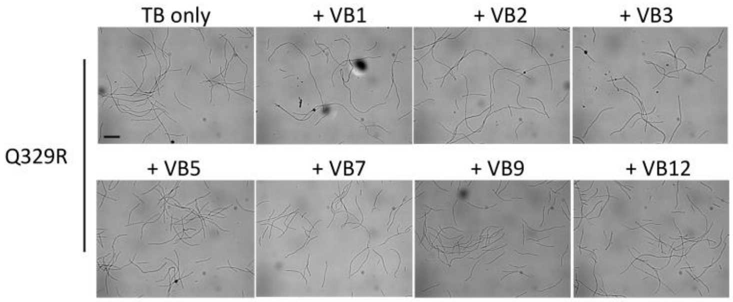

Supplementary Figure 8. Effects of various B-vitamins on CapV$_{Q329R}$-induced cell filamentation of *E. coli* MG1655. Addition of Vitamin B1 (thiamine, 0.5%), B2 (riboflavin, 0.5%), B3 (nicotinamide, 0.5%), B5 (pantothenate, 15 mg/ml), B7 (biotin, 100 ug/ml), B9 (folic acid, 80 ug/ml), and B12 (cobalamin, 200 ug/ml) in TB does not affect cell filamentation of *E. coli* MG1655 induced by CapV$_{Q329R}$ after 4 h at 37 °C. Bar, 10 µm. VC = pBAD28; CapV=wild type *capV* cloned in pBAD28; CapV$_{Q329R}$ = mutant CapV$_{Q329R}$ cloned in pBAD28.

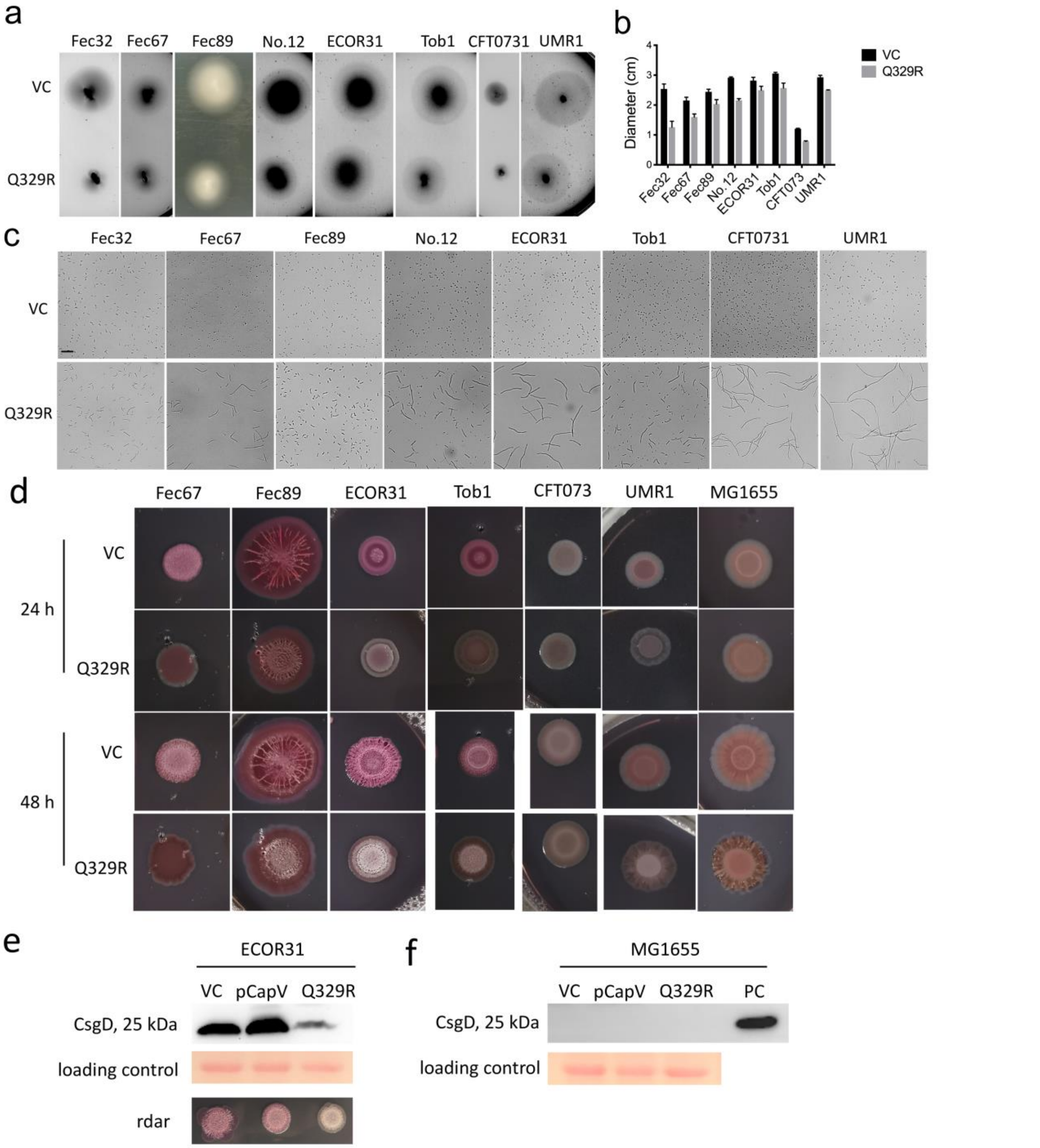

Supplementary Figure 9. Overexpression of CapV$_{Q329R}$ in *E. coli* MG1655 restricts apparent swimming motility, induces cell filamentation and alters rdar biofilm formation of a panel of commensal and UPEC *E. coli* strains and *S. typhimurium* UMR1. a, **b** 3 µl of OD_{600} = 5 cells were inoculated into soft agar plates containing 1% tryptone, 0.5% NaCl and 0.25% agar. The swimming diameter was measured after 6.5 h for *E. coli* ECOR31 and *S. typhimurium* UMR1, 8 h for *E. coli* Tob1, 9 h for *E. coli* No.12, Fec89, and CFT073, 10 h for *E. coli* Fec67 and 16 h for *E. coli* Fec32, respectively, at 37 °C. Bars represent mean values from three biologically independent replicates. **c** Cell filamentation of each strain was examined after 6 h of induction at 37 °C by a light microscope. Bar, 10 µm. VC = pBAD28; CapV$_{Q329R}$ = mutant CapV$_{Q329R}$ cloned in pBAD28. **d** Assessment of rdar morphotype expression on Congo red agar plates. Cells were grown on salt-free LB agar plates at 37 °C and observed after 24 h and 48 h. VC = pBAD28; CapV = wild type

capV cloned in pBAD28; $CapV_{Q329R}$ = mutant $CapV_{Q329R}$ cloned in pBAD28. (a-d) Commensal strains *E. coli* Fec32, Fec67, Fec89, ECOR31 and Tob1 and UPEC No.12 and CFT073 and the gastrointestinal pathogen *S. typhimurium* UMR1 were tested. **e** Rdar biofilm and CsgD expression of ECOR31 upon overexpression of CapV and $CapV_{Q329R}$. Cells were grown on salt-free LB agar plates at 37 °C, observed after 24 h and collected for CsgD expression detection by Western blot analysis after 16 h (addition of Congo red to the medium delays the development of the phenotype). As the loading control, the blot was stained with ponceau S after antigen detection. **f** CsgD expression of *E. coli* MG1655 upon overexpression of CapV and $CapV_{Q329R}$. Cells were grown on salt-free LB agar plates at 37 °C for 40 h and collected for CsgD expression detection by Western blot analysis. PC = *E. coli* TOB1 as a positive control for CsgD expression. As the loading control, the blot was stained with ponceau S after antigen detection. (e and f) VC = pBAD28; pCapV = wild type capV cloned in pBAD28; Q329R = mutant $CapV_{Q329R}$ cloned in pBAD28.

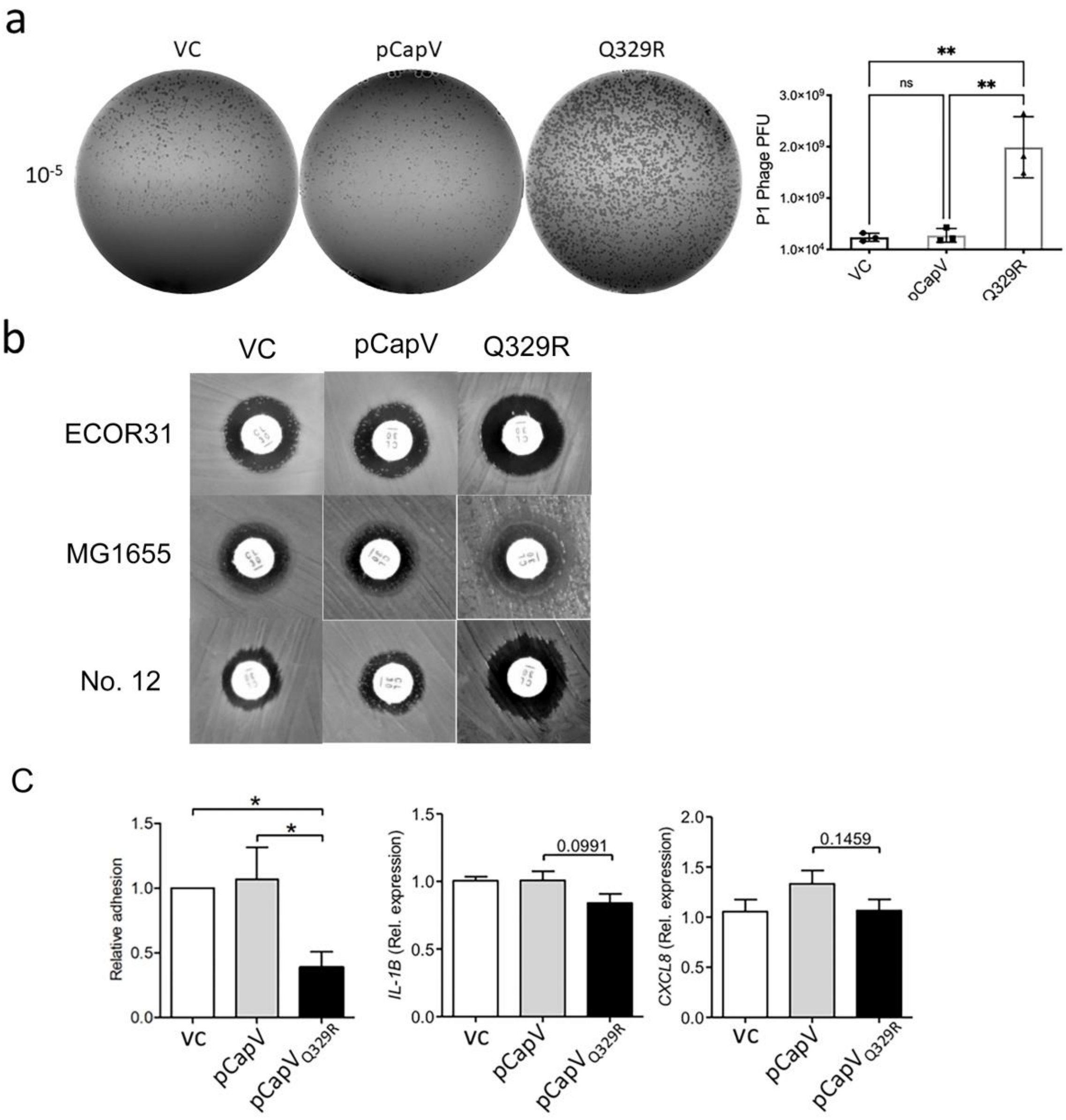

Supplementary Figure 10. Phage sensitivity and host cell interaction phenotypes mediated by pCapV$_{Q329R}$ in *E. coli* MG1655 and UPEC *E. coli* No. 12. a Plaque formation of bacteriophage P1 on *E. coli* MG1655 vector control and strains overexpressing CapV and CapV$_{Q329R}$. Two-layer agar plates with bacteriophage P1 overlay leading to plaque formation on the bacterial lawn. Graph displaying plaque-forming units (PFU) per milliliter; bar graph represents average of three biologically independent replicates. **b** Antimicrobial susceptibility of *E. coli* MG1655, ECOR31 and No. 12 against the cell wall targeting antibiotic cephalexin upon overexpression of CapV and CapVQ$_{329R}$. Discs contained 30 µg cephalexin with cells grown on Mueller Hinton agar with 0.1% L-arabinose and 25 µg/ml Cm. **c** Relative adhesion of *E. coli* No. 12 expressing CapV and CapV$_{Q329R}$ and relative steady state mRNA level of IL-1B and CXCL8 proinflammatory marker. VC = vector control pBAD28; pCapV = CapV cloned into pBAD28; pCapV$_{Q329R}$ = CapV$_{Q329R}$ cloned into pBAD28.

Supplementary tables

Table S1 Bacterial strains and plasmids used in this study.

**Strain or plasmid**	**Genotype or description**	**Reference or source**
E. coli strains		
K-12 derivatives		
MG1655	ATCC 700926	*(7)*
MG1655 Δ*sulA*	MG1655 Δ*sulA::kanR*	*(8, 9)*
BS001	MG1655 *ftsZ::gfp*	*(10)*
PB318	MG1655 *mCherry-minC (Km^r) yPet-zapB frt*	*(11)*
NEB 5-alpha	High efficiency chemically competent cells, T1 phage resistant and *endA* deficient	New England Biolabs
Top10	F- *mcrA* Δ(*mrr-hsdRMS-mcrBC*) φ80*lacZ*ΔM15 Δ*lacX74 nupG recA1 araD139* Δ(*ara-leu*)7697 *galE15 galK16 rpsL*(StrR) *endA1* λ-	Invitrogen
Wild type *E. coli* isolates		
ECOR31	Strain 31 of the ECOR *E. coli* reference collection	*(3)*
Tob1	Commensal human faecal isolate	*(12)*
Fec32	Commensal human faecal isolate	*(13)*
Fec67	Commensal human faecal isolate	*(13)*
Fec89	Commensal human faecal isolate	*(13)*
No.12	Wild-type pyelonephritis isolate from urine	(14)
CFT073	Wild-type pyelonephritis isolate	Laboratory collection
Salmonella enterica serovar Typhimurium		
UMR1	ATCC 14028 Nal^r, rdar at 28 °C	*(15)*
Vibrio cholerae		
V. cholerae C6706	*V. cholerae* O1 El Tor strain C6706	Laboratory collection
Plasmids		
pBAD28	Arabinose-regulated promoter; Amp^r; Cm^r	*(16)*
p*78901v*	pBAD28::*capV$_{Q329R}$ dncV* vc0180 vc0181**; Cm^r	*(1)*
pDncV	pBAD28::DncV-His$_6$; Cm^r	*(1)*
p79801	pBAD28::*dncV* vc0180 vc0181**; Cm^r	This study
pCapV	pBAD28::CapV; Cm^r	This study
pCapV_His	pBAD28:: CapV with C-terminal 6XHis tag; Cm^r	This study
pCapV$_{Q329R}$	pBAD28::CapV$_{Q329R}$; Cm^r	This study
pCapV$_{Q329R}$_His6	pBAD28::CapV$_{Q329R}$ with C-terminal 6XHis tag; Cm^r	This study
pCapV$_{Q329K}$	pBAD28::CapV$_{Q329K}$; Cm^r	This study
pCapV$_{Q329R/G24A/G25A}$	pBAD28::CapV$_{Q329R/G24A/G25A}$; Cm^r	This study
pCapV$_{Q329R/R27A}$	pBAD28::CapV$_{Q329R/R27A}$; Cm^r	This study
pCapV$_{Q329R/S64A}$	pBAD28::CapV$_{Q329R/S64A}$; Cm^r	This study

pCapV$_{Q329R/D197A}$	pBAD28::CapV$_{Q329R/D197A;}$ Cm^{r}	This study
pCapV$_{Q329R/D197A}$_His6	pBAD28::CapV$_{Q329R/D197A}$ with C-terminal 6XHis tag$_{;}$ Cm^{r}	This study
pCapV$_{Q329R/S33A}$_His6	pBAD28::CapV$_{Q329R/S33A}$ with C-terminal 6XHis tag$_{;}$ Cm^{r}	This study
pCapV$_{Q329R/S113/114A}$ _His6	pBAD28::CapV$_{Q329R/S113/114A}$ with C-terminal 6XHis tag$_{;}$ Cm^{r}	This study
pCapV$_{Q329R/S146A}$_His6	pBAD28::CapV$_{Q329R/S146A}$ with C-terminal 6XHis tag$_{;}$ Cm^{r}	This study
pCapV$_{Q329R/S177A}$_His6	pBAD28::CapV$_{Q329R/S177A}$ with C-terminal 6XHis tag$_{;}$ Cm^{r}	This study
pCapV$_{Q329R/S206A}$_His6	pBAD28::CapV$_{Q329R/S206A}$ with C-terminal 6XHis tag$_{;}$ Cm^{r}	This study

Table S2 Primers used in this study.

Primer	Sequence (5'-3')
Gene cloning	
VC0178-81-SacI-fw	GGCGAGCTCGCTATATTCTCTGGTTATGGGGTTTTCAATGTCTG[a]
VC0178-XbaI-rv	GCTCTAGATCAGAGTTTCTCCTGCGC[a]
VC0178-XbaI-RV	GCTCTAGATCAATGATGATGATGATGATGGAGTTTCTCCTGCGC[a]
VC0180-XmaI-rv	CCCCCCGGGTCATTTATGTTCATTACACACAGGGC[a]
DncV-SacI-fw	GGCGAGCTCGCAGGAGAAACTCTGATGCCTTG[a]
VC0178-81-SphI-rv	ACATGCATGCTTACTTCTCT CTTATAC[a]
VC0180-SacI-fw	GGCGAGCTCGATGGTAAGTGGCTGATGAAGGAC[a]
DHIII-RV	CCCAAGCTTCCGCATCCCGTCATCACTTGAT[a]
78_XbaI_fw	GCTCTAGAGCTATATTCTCTGGTTATGGGGTTTTCAATGTCTG[a]
78-SacI-rv	GGCGAGCTCTCAATGATGATGATGATGATGGAGTTTCTCCTGCGC[a]
Site-directed mutagenesis	
CapV-Q329K-Q5-fw	AAGGGGAAGAaAACTCGCTAC
CapV-Q329K-Q5-rv	GCGCGCAGGTTTTCAATG
CapV-Q329N-Q5-fw	AAGGGGAAGAaacCTCGCTACTG
CapV-Q329N-Q5-rv	GCGCGCAGGTTTTCAATG
CapV-G24/25A-Q5-fw	CTAAATGGTGcggcGGCCAGAGGGATGTTTAC[b]
CapV-G24/25A-Q5-rv	GCAGAGTAGCCGTACTCC
CapV-R27A-Q5-fw	TGGTGGGGCCgcgGGGATGTTTAC[b]
CapV-R27A-Q5-rv	CCATTTAGGCAGAGTAGC
CapV-S64A-Q5-fw	CGCAGGGACAgCGATAGGTGG[b]
CapV-S64A-Q5-rv	ATGAGGTCAAAGTAATCGCCAATTC
CapV-D197A-Q5-fw	TATTTTGCTGcgGGAGGTCTGGTC[b]
CapV-D197A-Q5-rv	AGAACCAAGGTCTTCACAATG
CapV-S33A-Q5-fw	GTTTACAATCgcgGTGCTTGCTGAGATTGAGAGGATACTTGC[b]
CapV-S33A-Q5-rv	ATCCCTCTGGCCCCACCA
CapV-S113/114A-fw	TCCCATTTATgcggcgAAACCACTTCGACAAACAATTG[b]
CapV-S113/114A-Q5-rv	GAAACAAGTGATTTAAGGAG
CapV-S146A-Q5-fw	AGTCAATCTTgcgACAGGAAAACCTC[b]
CapV-S146A-Q5-rv	GCCGGGACCATTACTCTC
CapV-S177A-Q5-fw	ACTCGCAACCgcgGCTGCACCGA[b]
CapV-S177A-Q5-rv	GCGGCATCAATCAATTTAAGTGCAC
CapV-S206A-Q5-fw	CAACAACCCCgcgTATATCGGATTTTTGGAAGTTTTCAG[b]
CapV-S206A-Q5-rv	GCGACCAGACCTCCATCA
d-xbaI-fw	ATGGTGAGCAAGGGCGAG
d-xbaI-rv	GAGTTTCTCCTGCGCGATTG
DncV-D129/131A-Q5-fw	tgcgGATGGCACCTACATGCCC[b]
DncV-D129/131A-Q5-rv	atcgCCATTTCCTGCCCCGGAT[b]
DncV-D197A-Q5-fw	ACACACATTGcgGTACCGATGTATG[b]

DncV-D197A-Q5-rv	TTTCTCCCGATAGATTTTG
VC0180-C91A-Q5-fw	AGGGTTCCTTgcgTATGTTGAGCAGATGGAAGCAGACTGGG[b]
VC0180-C91A-Q5-rv	TCCAGGGCGACATGGGGC
VC0180-C355A-Q5-fw	GTTGATAGGTgcgGGAACAATTGGAGGG[b]
VC0180-C355A-Q5-rv	GCTATACGCTTTGTTGAAAG
VC0181-E123A-Q5-fw	TATTTAGGCGcgTGGCATACACATC[b]
VC0181-E123A-Q5-rv	AACAAGAAAGCCATTTGAC
2401-fw	AGGGGAAGACaACTCGCTACTG[b]
2401-rv	TGCGCGCAGGTTTTCAAT
2805-fw	GACTTCATCAaATTCCAGCCTC[b]
2805-rv	ATCGCGTGCTTCATCTTC
3324-fw	GGCGGGCATCtGCGTTCAGTT[b]
3324-rv	GATACGTTTACAGCTTTCGTTGAACC
5496-fw	ATATCCTTGAgGGGGGGCGTT[b]
5496-rv	GTTCATTGTAAGTCCACTTTAACTC
Confirmatory primers	
pBAD30-fw	GTCTATAATCACGGCAGAAAAGTCCAC
pBAD30-rv	CTGTTTTATCAGACCGCTTCTGC
VC0178-inside-fw	GAAACGATGATCGGTGGTGAG
DncV-seq	TACATGCCCATGACGGTGTT
VC0180-inside-fw	CCCATCAGCACGCATTACATTTC
80-inside-fw2	GATACACCAGACCCTCATTG
VC0181-inside-rv	GGATGTGTATGCCATTCGCCTA

Underlined letters indicate [a]restriction sites, [b]mutated codons in miniscule letters.

Table S3 Plasmids used to assess the effect of mutations in p*78901v*.

**Strain or plasmid**	**Genotype or description**	**Source**
p*78901v*$_{D197A}$	pBAD28::*capV*$_{Q329R/D197A}$-*dncV*-vc0180-vc0181**	This study
D197A_78	pBAD28::*capV*$_{Q329R/D197A}$-*dncV*-vc0180-vc0181**	This study
D197A_79	pBAD28::*capV*$_{Q329R}$-*dncV*$_{K102R/D197A/L275P}$-*vc0180-vc0181**	This study
D129A/131A	pBAD28::*capV*$_{Q329R}$-*dncV*$_{K102R/D129A/D131A/L275P}$-*vc0180-vc0181**	This study
C91A	pBAD28::*capV*$_{Q329R}$-*dncV*-vc0180*$_{C91A}$-*vc0181**	This study
C355A	pBAD28::*capV*$_{Q329R}$-*dncV*-vc0180*$_{C355A}$-*vc0181**	This study
E123A	pBAD28::*capV*$_{Q329R}$-*dncV*-vc0180-vc0181*$_{E123A}$	This study
R329Q	pBAD28::*capV-dncV*-vc0180-vc0181**	This study
R102K	pBAD28::*capV*$_{Q329R}$-*dncV**$_{R102K}$-*vc0180-vc0181**	This study
P275L	pBAD28::*capV*$_{Q329R}$-*dncV**$_{P275L}$-*vc0180-vc0181**	This study
K6R	pBAD28::*capV*$_{Q329R}$-*dncV*-vc0180-vc0181*	This study

Table S4 Primers for qRT-PCR of bacterial genes.

Primer	Sequence (5'-3')
flhD-qRT-fw	ATCAAATCCCGTCTCGATG
flhD-qRT-rv	GTTTGACCATCATTCGCAC
fliA-qRT-fw	AATAACAGCCAGCTCTTCTC
fliA-qRT-rv	TCCAGTAGTTGTTGTAGCGG
fliC-qRT-fw	ACTACTGGTGCTGTTTCTG
fliC-qRT-rv	TCGACCACTTCTGTTTTGC
ftsZ-qRT-fw	GAAGTGGCAAAAGATTTGGG
ftsZ-qRT-rv	CTTGGACAGTTCAGTGATCC
recA-qRT-fw	GACTGCCTGGCTGAAAGA
recA-qRT-rv	GAGAAATCCGGCGTTGAG
rpsV-qRT-fw	CAGGCACGTCATATTCTTG
rpsV-qRT-rv	GTTGGGTTATTTACCACGC

Supplementary Figures

Additional file types that cannot be embedded into the Word file:

Movie S1. Movie of *E. coli* MG1655 cells upon overexpression of CapV wild type and $CapV_{Q329R}$ induced by 0.1% L-arabinose at 37 °C in TB medium while being examined after 4 and 6 h under a microscope. (a) *E. coli* MG1655 vector control pBAD28, 4h; (b) *E. coli* MG1655 pCapV, 4h; (c) *E. coli* MG1655 pCapVQ329R, 4h; (d) *E. coli* MG1655 pBAD28, 6h; (e) *E. coli* MG1655 pCapV, 6h; (f) *E. coli* MG1655 pCapVQ329R, 6h. VC = pBAD28; CapV = wild type capV cloned in pBAD28; $CapV_{Q329R}$ = mutant $CapV_{Q329R}$ cloned in pBAD28.

Movie S2. *E. coli* MG1655 cells examined under a light microscope 22 h after induction of $CapV_{Q329R}$ production by 0.1% (a) and 0.2% (b) L-arabinose in TB medium at 37 °C. $CapV_{Q329R}$ = mutant $CapV_{Q329R}$ cloned in pBAD28.

Movie S3. Time-lapse analysis of *E. coli* MG1655 *mCherry-minC* expressing cells with pBAD28 vector control (a), pCapV (b) and $pCapV_{Q329R}$ (c). VC = pBAD28; CapV = wild type CapV cloned in pBAD28; $CapV_{Q329R}$ = mutant $CapV_{Q329R}$ cloned in pBAD28.

References

1. Li F, *et al.* (2019) DncV synthesizes cyclic GMP-AMP and regulates biofilm formation and motility in *Escherichia coli* ECOR31. *mBio* 10, e02492-02418.
2. Davies BW, Bogard RW, Young TS, & Mekalanos JJ (2012) Coordinated regulation of accessory genetic elements produces cyclic di-nucleotides for *V. cholerae* virulence. *Cell* 149, 358-370.
3. Schubert S, Dufke S, Sorsa J, & Heesemann J (2004) A novel integrative and conjugative element (ICE) of *Escherichia coli*: the putative progenitor of the *Yersinia* high-pathogenicity island. *Mol Microbiol* 51, 837-848.
4. Severin GB, *et al.* (2018) Direct activation of a phospholipase by cyclic GMP-AMP in El Tor *Vibrio cholerae*. *Proc Natl Acad Sci* 115, E6048-E6055.
5. Thompson JD, Higgins DG, & Gibson TJ (1994) CLUSTAL W: improving the sensitivity of progressive multiple sequence alignment through sequence weighting, position-specific gap penalties and weight matrix choice. *Nucleic Acids Res* 22, 4673-4680.
6. Robert X & Gouet P (2014) Deciphering key features in protein structures with the new ENDscript server. *Nucleic Acids Res* 42(Web Server issue), W320-324.
7. Fang X & Gomelsky M (2010) A post-translational, c-di-GMP-dependent mechanism regulating flagellar motility. *Mol Microbiol* 76, 1295-1305.
8. Harms A, Fino C, Sørensen MA, Semsey S, & Gerdes K (2017) Prophages and growth dynamics confound experimental results with antibiotic-tolerant persister cells. *mBio* 8, e01964-01917.
9. Goormaghtigh F & Van Melderen L (2019) Single-cell imaging and characterization of *Escherichia coli* persister cells to ofloxacin in exponential cultures. *Sci Adv* 5, eaav9462.
10. Söderström B, *et al.* (2014) Disassembly of the divisome in *Escherichia coli*: evidence that FtsZ dissociates before compartmentalization. *Mol Microbiol* 92, 1-9.
11. Bisicchia P, Arumugam S, Schwille P, Sherratt D, & Gottesman S (2013) MinC, MinD, and MinE drive counter-oscillation of early-cell-division proteins prior to *Escherichia coli* septum formation. *mBio* 4, e00856-13.
12. Cimdins A, *et al.* (2017) Alterations of c-di-GMP turnover proteins modulate semi-constitutive rdar biofilm formation in commensal and uropathogenic *Escherichia coli*. *MicrobiologyOpen* 6, e00508.
13. Bokranz W, Wang X, Tschäpe H, & Römling U (2005) Expression of cellulose and curli fimbriae by *Escherichia coli* isolated from the gastrointestinal tract. *J Med Microbiol* 54, 1171-1182.
14. Kai-Larsen Y, *et al.* (2010) Uropathogenic *Escherichia coli* modulates immune responses and its curli fimbriae interact with the antimicrobial peptide LL-37. *PLoS Pathog* 6, e1001010.
15. Römling U, Sierralta WD, Eriksson K, & Normark S (1998) Multicellular and aggregative behaviour of *Salmonella typhimurium* strains is controlled by mutations in the *agfD* promoter. *Mol Microbiol* 28, 249-264.
16. Guzman LM, Belin D, Carson MJ, & Beckwith J (1995) Tight regulation, modulation, and high-level expression by vectors containing the arabinose PBAD promoter. *J Bacteriol* 177, 4121-4130.

Additional data

Uncropped gels and blots

Fig. 1f

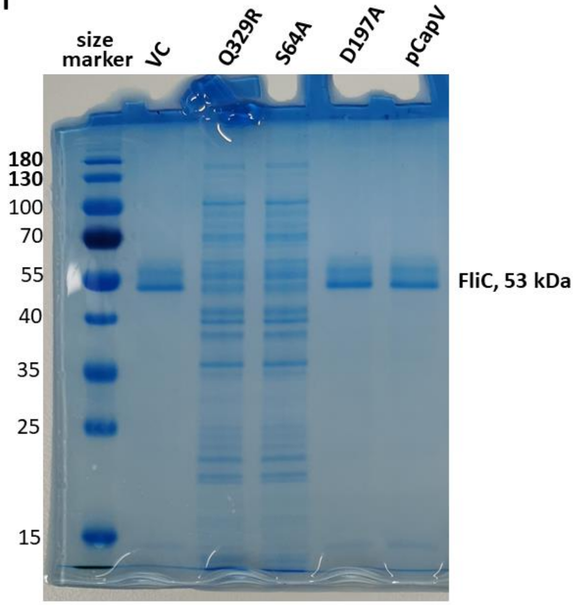

Fig. 1g

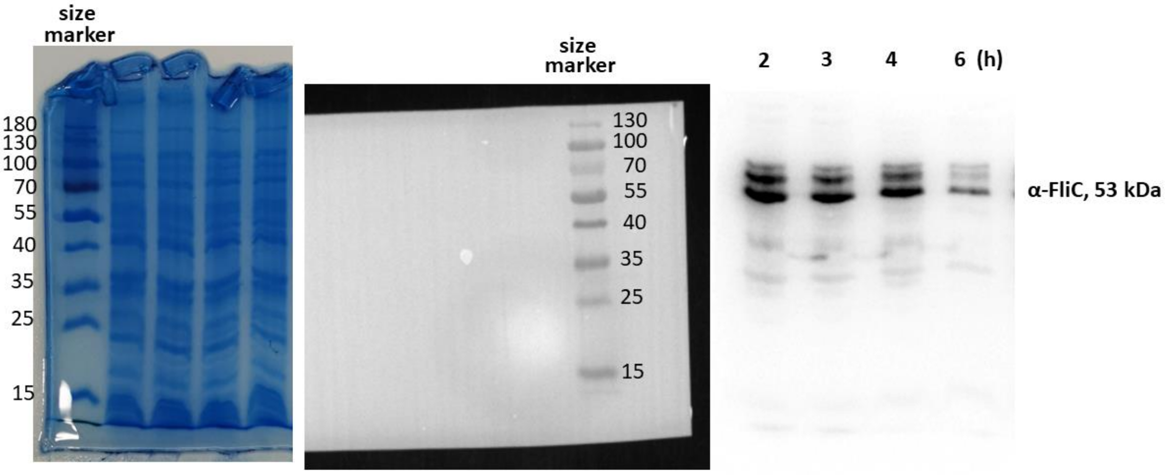
size marker
180
130
100
70
55
40
35
25
15
size marker
130
100
70
55
40
35
25
15
2
3
4
6 (h)
α-FliC, 53 kDa

Fig. 2f

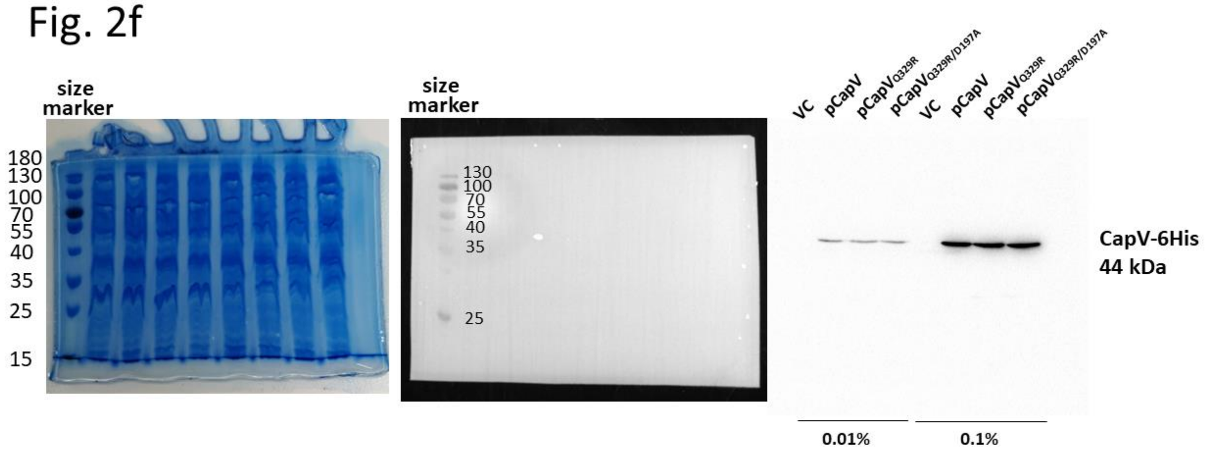
size marker
180
130
100
70
55
40
35
25
15
size marker
130
100
70
55
40
35
25
VC
pCapV
pCapV$_{Q329R}$
pCapV$_{Q329R/D197A}$
VC
pCapV
pCapV$_{Q329R}$
pCapV$_{Q329R/D197A}$
CapV-6His
44 kDa
0.01%
0.1%

Fig. 8c

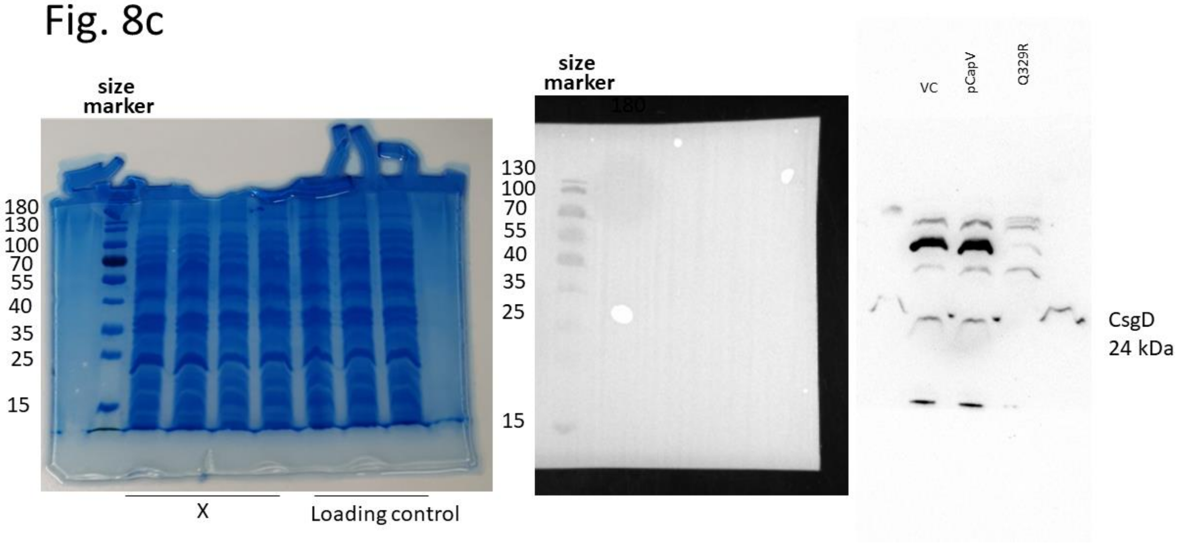

size marker
180
130
100
70
55
40
35
25
15
X
Loading control
size marker
180
130
100
70
55
40
35
25
15
VC
pCapV
Q329R
CsgD
24 kDa

Fig. S9e

E. coli ECOR31

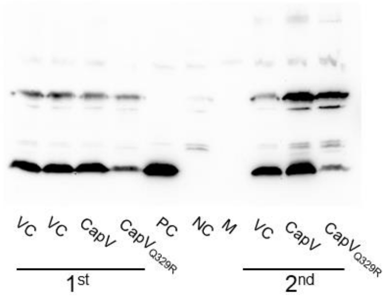

Fig. S9f

E. coli MG1655

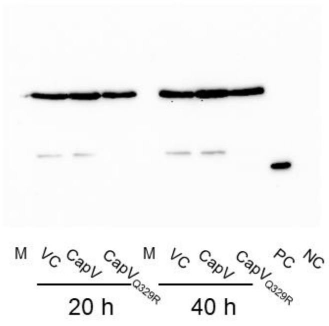

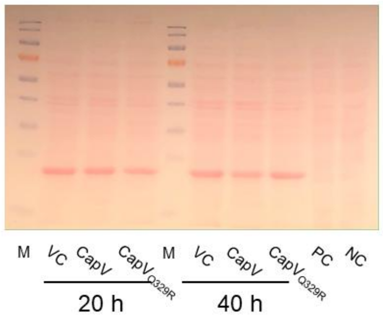

Supplement: Supplementary file 1 — Supplementary Information [file 41522_2022_294_MOESM1_ESM.pdf]
